# Body mass index and psychiatric disorders: a Mendelian randomization study

**DOI:** 10.1038/srep32730

**Published:** 2016-09-07

**Authors:** Fernando Pires Hartwig, Jack Bowden, Christian Loret de Mola, Luciana Tovo-Rodrigues, George Davey Smith, Bernardo Lessa Horta

**Affiliations:** 1 Postgraduate Program in Epidemiology, Federal University of Pelotas, Pelotas, Brazil; 2 MRC Integrative Epidemiology Unit, University of Bristol, Bristol, UK; 3 MRC Biostatistics Unit, University of Cambridge, Cambridge, UK

## Abstract

Obesity is a highly prevalent risk factor for cardiometabolic diseases. Observational studies suggest that obesity is associated with psychiatric traits, but causal inference from such studies has several limitations. We used two-sample Mendelian randomization methods (inverse variance weighting, weighted median and MR-Egger regression) to evaluate the association of body mass index (BMI) with three psychiatric traits using data from the Genetic Investigation of Anthropometric Traits and Psychiatric Genomics consortia. Causal odds ratio estimates per 1-standard deviation increment in BMI ranged from 0.88 (95% CI: 0.62; 1.25) to 1.23 (95% CI: 0.65; 2.31) for bipolar disorder; 0.93 (0.78; 1.11) to 1.41 (0.87; 2.27) for schizophrenia; and 1.15 (95% CI: 0.92; 1.44) to 1.40 (95% CI: 1.03; 1.90) for major depressive disorder. Analyses removing potentially influential SNPs suggested that the effect estimates for depression might be underestimated. Our findings do not support the notion that higher BMI increases risk of bipolar disorder and schizophrenia. Although the point estimates for depression were consistent in all sensitivity analyses, the overall statistical evidence was weak. However, the fact that SNP-depression associations were estimated in relatively small samples reduced power to detect causal effects. This should be re-addressed when SNP-depression associations from larger studies become available.

Obesity is a major public health concern with well-established risk-increasing effects on cardiometabolic diseases^1^. Given its high prevalence worldwide^1^, investigating if obesity influences additional diseases is relevant for understanding the range of its health consequences.

Psychiatric disorders are one of the main causes of years lived with disability globally^2^. There is considerable evidence suggesting an association between obesity and psychiatric disorders, including depression^3^,^4^, bipolar disorder^5^,^6^ and schizophrenia^7^,^8^. Reverse causality could be one of the explanations for this association because increase in body weight is a side effects of some anti-psychotic medications^6^,^9^. Besides treatment, biological, psychological, and sociodemographic variables related to psychiatric disorders may affect lifestyle factors such as physical activity and diet and thus lead to obesity^10^,^11^.

Cohort studies provide support that obesity both predicts and can be predicted by depression^3^,^12^,^13^ and bipolar disorder^14^. Moreover, higher frequencies of obesity measures were reported in first episode and/or medication-naive schizophrenia patients^15^,^16^, although not universally^17^,^18^. A recent instrumental variable analysis supported the hypothesis that obesity influences depression^19^.

Most of the evidence regarding the association of obesity with psychiatric disorders comes from observational studies, which present several limitations for causal inference, including residual confounding, measurement error and reverse causation^20^,^21^. Using genetic variants as instrumental variables for modifiable disease risk factors or exposures (ie, Mendelian randomization) contributes to overcome such limitations given Mendel’s laws, the fact that germline genetic variants are determined at conception and the general lack of association between genetic variants and common confounders of observational associations^21^,^22^,^23^.

Mendelian randomization relies on assuming that any association between the genetic instrument(s) and the health outcome is entirely mediated by the exposure (ie, vertical pleiotropy)^21^,^22^,^23^. However, the polygenic nature of complex traits increases the probability of existing biological links between exposure-associated variants and the outcome not mediated by the exposure itself (ie, horizontal pleiotropy). Indeed, the largest genome-wide association study (GWAS) of body mass index (BMI) to date identified variants implicated in biological pathways related to the central nervous system^24^, thus potentially complicating Mendelian randomization involving BMI and psychiatric disorders.

Previous Mendelian studies on this topic yielded inconsistent findings. However, such studies have some limitations, including using only *FTO* and *MC4R* variants as genetic instruments^25^,^26^, both of which are pleiotropic loci^27^,^28^ that possibly violate Mendelian randomization assumptions^25^,^29^,^30^. Another limitation is that the studies were performed in the single-sample context^25^,^26^,^29^,^31^,^32^, which renders the results prone to bias towards the observational (and possibly confounded) estimate if the genetic instrument is weakly associated with BMI^33^,^34^.

Currently, many GWAS consortia make summary-level results freely available. Such data can be used to obtain causal effect estimates based on multiple single nucleotide polymorphisms (SNPs) using the inverse-variance weighting (IVW) method^35^. This is likely to improve statistical power because SNP-exposure and SNP-outcome associations are typically estimated in large samples. Moreover, the recently proposed MR-Egger regression^36^ and weighted median^37^ methods can be used in summary data Mendelian randomization investigations as sensitivity analyses to detect and (at least partially) account for violations of instrumental variable assumptions. MR-Egger causal effect estimates are consistent even if all instruments are invalid, as long as SNP-exposure and direct (ie, not solely mediated by the exposure) SNP-outcome associations are independent. The weighted median requires that at least 50% of the information come from valid instruments; if this is satisfied, its causal effect estimate is consistent regardless of the type of horizontal pleiotropy in the invalid instruments (see Materials and Methods section for details). We aimed at estimating the causal effect of BMI on three psychiatric traits using these three Mendelian randomization methods in a two-sample framework.

## Results

### Estimation of causal effects of BMI on schizophrenia using Mendelian randomization

**Figure 1:**
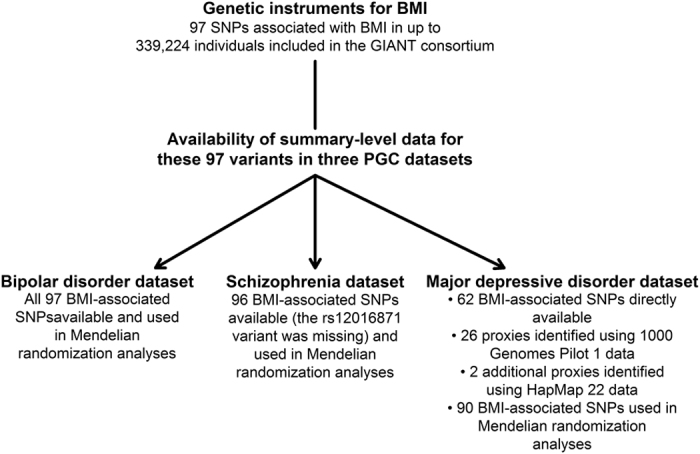
Flowchart depicting the selection process of the genetic instruments for body mass index. BMI: Body mass index. SNP: Single nucleotide polymorphism. GIANT: Genetic Investigation of Anthropometric Traits consortium. PGC: Psychiatric Genomics Consortium.

A flowchart depicting the selection process of the genetic variants used in Mendelian randomization analysis is shown in Fig. 1. Table 1 describes each psychiatric dataset. SNP-BMI F-statistics in the Genetic Investigation of Anthropometric Traits (GIANT) consortium dataset were similar across the variants available in each psychiatric dataset, with mean and median values about 56 and 34, respectively. However, approximate SNP-BMI F-statistics (ie, instrument strength) in psychiatric datasets were considerably different: mean and median values were ~3 and ~2, respectively, for bipolar and major depressive disorders, and 14.4 and 9.0 for schizophrenia. Tetrachoric correlations between GIANT and each PGC datasets were very close to zero, suggesting that there was, at most, little sample overlap (see Materials and Methods for details).

Mendelian randomization results for each psychiatric disorder are shown in Table 2. For bipolar disorder, odds ratio of 0.90 (95% CI: 0.69; 1.16) and 0.88 (95% CI: 0.62; 1.25) per 1-standard deviation (SD) increment in BMI were obtained using the IVW and weighted median methods. These estimates were directionally inconsistent with regular and Simulation Extrapolation (SIMEX)-corrected MR-Egger estimates of 1.23 (95% CI: 0.65; 2.31) and 1.26 (95% CI: 0.63; 2.52), respectively. Schizophrenia presented a similar pattern, with IVW and weighted median odds ratio of 0.98 (0.80; 1.19) and 0.93 (0.78; 1.11), respectively, while regular and SIMEX-corrected MR-Egger estimates were 1.41 (0.87; 2.27) and 1.46 (0.86; 2.47), respectively. Regarding major depressive disorder, all methods yielded directionally consistent estimates: IVW, weighted median, regular and SIMEX-corrected MR regression odds ratio were (respectively) 1.15 (95% CI: 0.92; 1.44), 1.40 (95% CI: 1.03; 1.90), 1.28 (95% CI: 0.74; 2.24) and 1.33 (95% CI: 0.72; 2.47). 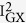 (which measures regression dilution bias in MR-Egger regression)^38^ was 88.6%, 88.2% and 87.9% for bipolar disorder, major depressive disorder and schizophrenia, respectively, suggesting an attenuation of the causal effect estimates of about 12% due to regression dilution bias (which can be seen comparing regular and SIMEX-corrected MR-Egger results). All MR-Egger intercepts were close to 1.00 and none achieved conventional statistical significance levels, suggesting that there was no strong directional horizontal pleiotropy under the InSIDE assumption.

### Sensitivity analyses removing influential instruments and within subgroups of biological categories

**Figure 2:**
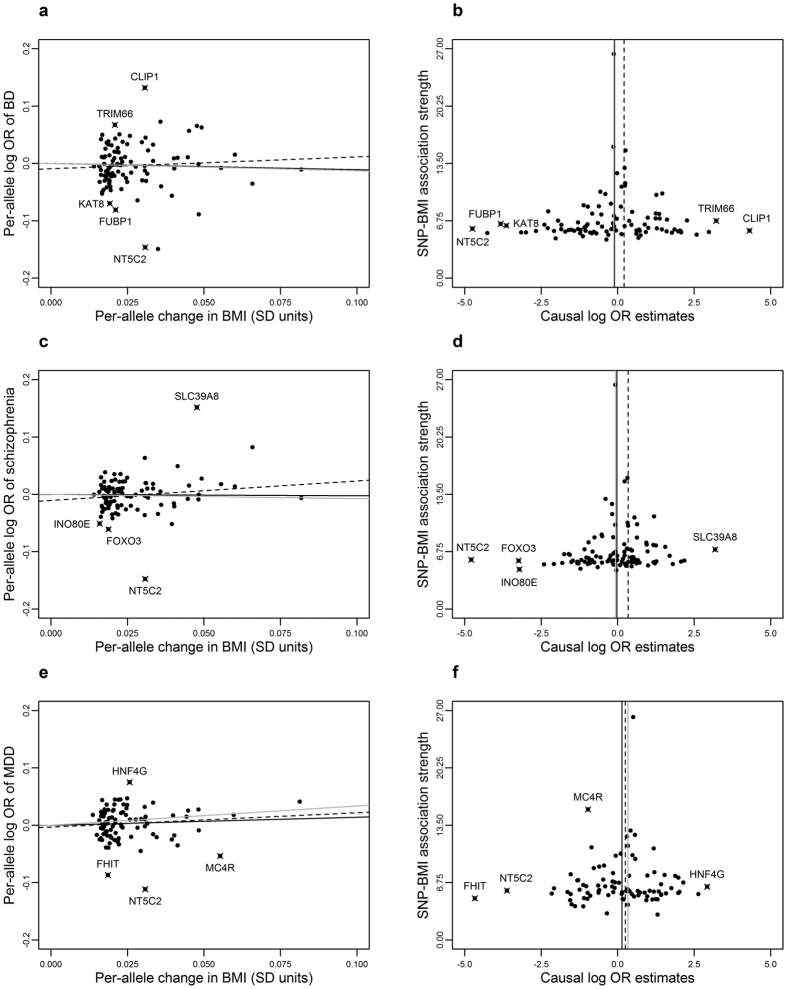
SNP-BMI and SNP-psychiatric associations for up to 97 BMI-associated SNPs identified by the GIANT consortium. Influential SNPs were marked with an “X” and labelled using the correspondent gene locus. SNPs were classified as influential using statistical tests based on studentized residuals and Cook’s distance (see Materials and Methods for details). Left column: scatter plots of associations between SNPs and (**a**) bipolar disorder (**b,d,c**) schizophrenia and (**e**) major depressive disorder (MDD) against SNP-BMI associations. IVW, MR-Egger and weighted median estimates are indicated in solid, dashed and grey lines, respectively. Right column: funnel plots of the absolute value of the *t*-statistic of SNP-BMI association (ie, instrument strength) against individual-SNP ratio estimates in log odds ratio of (**b**) bipolar disorder, (**d**) major depressive disorder and (**f**) schizophrenia. IVW, MR-Egger and weighted median estimates are indicated in solid, dashed and grey lines, respectively.

SNPs were classified as influential using statistical tests based on studentized residuals and Cook’s distance (see Materials and Methods for details). The following SNPs – rs number (gene locus) – were classified as influential: rs4256980 (*TRIM66*), rs12401738 (*FUBP1*), rs9925964 (*KAT8*), rs11191560 (*NT5C2*), rs11057405 (*CLIP1*) in bipolar disorder; rs13107325 (*SLC39A8*), rs11191560 (*NT5C2*), rs9400239 (*FOXO3*) and rs4787491 (*INO80E*) in schizophrenia; and rs571312 (*MC4R*), rs1462433 (*HNF4G*), rs6785875 (*FHIT*) and rs11191560 (*NT5C2*) in major depressive disorder (Fig. 2). Removing influential SNPs made virtually no difference in bipolar disorder results. Regarding schizophrenia, removing influential SNPs attenuated (and increased precision) regular and SIMEX-corrected (respectively) MR-Egger regression odds ratio to 1.22 (95% CI: 0.83; 1.81) and 1.25 (95% CI: 0.81; 1.92) per 1-SD increment in BMI. The magnitude of all major depressive disorder estimates increased, ranging from 1.25 (95% CI: 1.02; 1.52) to 1.60 (95% CI: 0.93; 2.75) using IVW and SIMEX-correct MR-Egger, respectively.

When dividing SNPs into neuronal-related vs. non-neuronal-related subgroups, the results were generally similar between subgroups (Table 3). Regarding bipolar disorder, in all cases the IVW and weighted median estimates were directionally inconsistent with MR-Egger results. Regular and SIMXEX-corrected MR-Egger odds ratio estimates were weaker in the neuronal-related (1.19 [95% CI: 0.54; 2.60] and 1.19 [0.51; 2.75] per 1-SD increment in BMI, respectively) than in the remaining SNPs (1.33 [95% CI: 0.38; 4.68] and 1.42 [95% CI: 0.32; 6.34], respectively). This difference was more evident after excluding influential SNPs, but in both cases confidence intervals of one subgroup largely included the point estimate of the other subgroup. In schizophrenia, the odds ratio estimates were also inconsistent, especially in the non-neuronal subgroup. Again, MR-Egger estimates were stronger in the non-neuronal subgroup, although such difference was attenuated after excluding influential SNPs and confidence intervals were wide. All major depressive disorder estimates were directionally consistent and similar between neuronal-related and non-neuronal subgroups. Excluding influential SNPs increased the estimates (especially IVW and MR-Egger ones).

IVW estimates for each outcome when SNPs belonging to a given biological category were removed are shown in Table 4. Regarding bipolar disorder, all but one of the IVW odds ratio estimates were directionally consistent, ranging from 0.75 (95% CI: 0.55; 1.03) to 0.98 (95% CI: 0.75; 1.28) per 1-SD increment in BMI. The exception was an estimate of 1.06 (95% CI: 0.81; 1.41), obtained after excluding SNPs prioritized by annotation tools, but that do not belong to a well-defined biological category (referred to as an “unspecified” biological category). Schizophrenia estimates were more heterogeneous, with 10 being smaller than 1 (ranging from 0.92 to 0.99) and six being larger than or equal to 1 (ranging from 1.00 to 1.04). Conversely, all major depressive disorder odds ratio estimates were directionally consistent and ranged from 1.06 (95% CI: 0.81; 1.39) to 1.29 (95% CI: 1.01; 1.64).

### Sensitivity analyses based on random effects meta-regression

Values of the meta-analytical measures of heterogeneity *τ*^*2*^ and *I^2^* (not 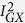) in the individual-SNP ratio estimates were 0.45 and 29.1% (*P* = 0.005) for bipolar disorder, 0.49 and 68.8% (P = 9.9 × 10^−24^) for schizophrenia, and 0.20 and 18.4% (*P* = 0.073) for major depressive disorder. In a random effects meta-regression model, including an indicator variable of influentiality status reduced *τ*^*2*^ and *I^2^* values of major depressive disorder ratio estimates to 0.14 and 13.4%, respectively, with an adjusted-R^2^ value (which indicates the amount of heterogeneity explained by the moderators) of 29.3% (*P* = 0.028), and the test of residual between-instruments heterogeneity yielded *P* = 0.152. Regarding bipolar disorder and schizophrenia, the same procedure had a substantially smaller influence, with adjusted-R^2^ values of 1.1% (*P* = 0.015; test of residual between-study heterogeneity *P* = 2.4 × 10^−23^) and 2.4% (*P* = 0.193; test of residual between-study heterogeneity *P* = 0.006), respectively. When an indicator of belonging to a neuronal-related biological category was used instead of influentiality status, all adjusted-R^2^ values were 0%.

The results of the forward selection process of biological moderators of individual-SNP ratio estimates using random effects meta-regression are shown in Supplementary Table S1. Adopting a P ≥ 0.05 stopping criterion resulted in the selection of two moderators for each outcome: unspecified and endocytosis/exocytosis categories for bipolar disorder; neurotransmission and lipid-related for schizophrenia; and lipid-related and glucose homeostasis/diabetes for major depressive disorder. Adjusted-R^2^ and residual *I^2^* values for the selected moderators together were 32.9% and 21.5% for bipolar disorder, 8.3% and 66.7% for schizophrenia and 68.8% and 6.5% for major depressive disorder. Mendelian randomization odds ratio estimates excluding each and both of the selected biological categories are shown in Supplementary Table S2. Again, only major depressive disorder presented directionally consistent estimates in all Mendelian randomization methods and SNP subgroups.

## Discussion

In the present study, we evaluated the association between BMI-associated SNPs and three psychiatric disorders by Mendelian randomization using summary-level data. Only major depressive disorder presented consistent causal effect estimates using the three Mendelian randomization methods and in all sensitivity analyses. The fact that removing influential variants increased, rather than attenuated, the odds ratio estimates is also reassuring because it suggests that the true causal effect might be greater than that estimated using all variants simultaneously. However, the overall statistical evidence for any meaningful associations was weak.

Our findings suggest that the commonly positive association between obesity and psychiatric disorders reported in observational studies^3^,^4^,^5^,^6^,^7^,^8^ may not correspond to a causal risk-increasing effect (especially for bipolar disorder and schizophrenia). Such associations may have been driven by phenomena such as residual confounding, due to common causes imperfectly accounted for at study design and/or analysis; or reverse causation, due to, for example, side effects of anti-psychotic medication. Even though associations of obesity with later depression and bipolar disorder have been reported in cohort studies^3^,^12^,^13^,^14^, it is still possible that reverse causation occurred due to effects (not related to medication usage) of pre-clinical psychiatric disorders on weight gain if such effects exist. Effects of pre-clinical psychiatric diseases have been recently detected in a longitudinal study where genetic predisposition to schizophrenia was associated with non-participation over time^39^.

Mendelian randomization studies on the association between obesity and psychiatric disorders are scarce, with no studies for bipolar disorders and schizophrenia. A positive association between BMI instrumented using the *FTO* variant rs1421085 and common mental disorders was detected in the British Whitehall II study^25^. However, adiposity measures instrumented by *FTO* and *MC4R* variants were inversely associated with psychological distress in a much larger Danish cohort^26^. These findings must be interpreted cautiously since there is evidence that *FTO*^27^ and *MC4R*^28^ are pleiotropic, in accordance with suggestions that *FTO* might not be a valid instrument for BMI when mental disorders are the outcome^25^,^29^. In our study, the *MC4R* variant rs571312 (and others, but not *FTO*) was identified as an influential (and potentially invalid) instrument in major depressive disorder analysis, but not in the remaining outcomes. In the Young Finns cohort, BMI instrumented by a 31-SNP allele score was positively associated with depressive symptoms^31^. However, two other studies using a similar genetic instrument failed to detect any association with depression-related outcomes, with risk-decreasing point estimates^29^,^32^. Our study extends Mendelian randomization analysis of the causal effects of BMI on psychiatric outcomes by using the more recently described set of 97 BMI-associated variants.

Obesity and psychiatric disorders may share several dysregulated physiological pathways, including inflammation^40^. Elevated inflammation is a potential cause of psychiatric disorders^41^ since positive associations between inflammatory markers and later psychiatric-related outcomes have been reported^13^,^41^. Given the well-defined role of obesity in inflammation, the later could be a mediator between obesity and psychiatric disorders. Indeed, a study among older English adults reported that C-reactive protein mediated about 20% of the longitudinal association between obesity and depressive symptoms^13^. However, there are only a few longitudinal studies evaluating this association^41^ and a large Mendelian randomization study did not suggest a causal association between C-reactive protein and depression^42^. The latter (assuming that higher BMI raises C-reactive protein levels) is in accordance with our inconsistent findings regarding the association of genetically elevated BMI with bipolar disorder and schizophrenia and the weak statistical evidence regarding the association with depression. Nevertheless, further studies are required to understand the role of inflammation and other biological pathways shared by obesity and psychiatric disorders in the latter.

European ancestry was predominant in all datasets, which increases the plausibility of the assumption that the two datasets are samples from the same or comparable populations. Regarding power, two-sample Mendelian randomization power depends more on the precision of SNP-outcome than SNP-exposure associations^35^. SNP-outcome associations used in this study were estimated in relatively small samples, except for schizophrenia. Sample size differences resulted in considerably different approximate SNP-BMI F-statistics across psychiatric datasets. Although SNP-BMI associations used in the analyses were obtained from GIANT, this difference suggests that, for bipolar and major depressive disorders, SNP-outcome associations were too imprecise, which is likely to decrease power. Indeed, in spite of the consistency across Mendelian randomization methods and sensitivity analyses, causal effect estimates for major depressive disorder – especially when using MR-Egger – had wide confidence intervals and in some cases failed to achieve conventional statistical significance levels. On the other hand, given the aforementioned consistency and the fact that sensitivity analyses suggested even stronger causal effect estimates, it is possible that there will be adequate statistical power to detect causal effects once more precise SNP-major depressive disorder estimates are available.

It is impossible to prove empirically whether Mendelian randomization results mostly reflect causal effects of the exposure or violations of instrumental variable assumptions. In the present study, three Mendelian randomization methods – each with different assumptions regarding horizontal pleiotropy – were used, and all of them were consistent regarding major depressive disorder, but not when analyzing bipolar disorder and shcizophrenia. Moreover, both MR-Egger regression and the weighted median approach (which are more robust against bias due to horizontal pleiotropy than IVW) point estimates were stronger than the IVW one. It was also reassuring that major depressive disorder presented the smallest heterogeneity in individual-SNP ratio estimates measured using the conventional *I*^2^ statistic, and that excluding four influential SNPs increased the magnitude of the causal effect estimates and further attenuated the *I*^2^ statistic.

In general, our findings do not corroborate the notion that BMI has a causal effect on bipolar disorder or schizophrenia. Regarding major depressive disorder, although the point estimates were consistent across a range of analyses, the overall statistical evidence was weak. Re-addressing this research question once SNP-depression associations from larger GWAS become available would be warranted to obtain more precise Mendelian randomization estimates (especially with respect to MR-Egger regression). Given the high prevalence of both obesity and depression worldwide, understanding the mechanisms underlying associations between BMI and depression, with identification and quantification of causal effects, is of public health relevance. Analyses involving schizophrenia were less prone to power issues because SNP-schizophrenia associations were estimated in a relatively large sample. Bipolar disorder, similarly to major depressive disorder, require further investigation once more precise SNP-outcome associations are available.

## Materials and Methods

### Data sources

The final datasets were provided in Supplementary Tables S3–S6.

#### Body mass index

Locke and colleagues’, under the GIANT consortium, identified 97 BMI-associated single nucleotide polymorphisms (SNPs)^24^. SNP-BMI linear regression coefficients and standard error estimates were obtained from an analysis of up to 322,154 European ancestry individuals assuming additive genetic effects (https://www.broadinstitute.org/collaboration/giant/index.php/GIANT_consortium_data_files).

The outcome was obtained by applying an inverse normal transformation to BMI residuals on age and age^2^ (in addition to relevant study-specific covariates such as ancestry-informative principal components). In studies of unrelated individuals, residuals were calculated within sex and (when relevant) case/control strata. In family studies, residuals were sex-adjusted rather than sex-specific.

To investigate the biological function of the 97 BMI-associated variants, Locke and colleagues assigned, for each SNP, all genes within 500 kb and *r*^2^ > 0.2. For variants without genes mapping to this interval, the nearest gene was used. This resulted in 405 genes, which were annotated based on a manual literature review using several venues. Through this process, those 405 genes were manually curated into 25 biological categories containing at least three genes, to which each of the 97 BMI-associated variants were assigned (see Locke and colleagues^24^ for details). We only considered the 16 categories that contained at least nine (~10%) SNPs (Supplementary Table 5S). Those categories substantially overlapped: four of them (“neuronal developmental processes”, “neurotransmission”, “hypothalamic expression and regulatory function” and “neuronal expression”) were neuronal-related and 62 SNPs were present in two or more categories. It is also noteworthy that those categories were aimed at providing insights into the biological processes implicated in obesity based on genetic associations rather than a detailed biological description of each SNP. Such biological categorizations are of approximate and provisional nature, changing over time as new data emerges^43^. The biological categories were used in the present work for sensitivity analyses purposes only (as described in the “Statistical analyses” section).

#### Psychiatric disorders

Log odds ratio and standard error estimates of SNPs-psychiatric disorders associations were obtained from the Psychiatric Genomics Consortium (PGC) (http://www.med.unc.edu/pgc/downloads), which performed logistic regression adjusting for ancestry-informative principal components and assuming an additive effect.

##### Bipolar disorder

Sklar and colleagues’ – under the PGC Bipolar Disorder Working Group – performed SNP-bipolar disorder associations on 7,481 cases and 9,250 controls of European descent^44^. All 97 BMI-associated SNPs were available. After harmonizing effect and non-effect alleles between SNP-bipolar disorder and SNP-BMI datasets, the Pearson correlation coefficient between effect allele frequencies was > 0.999.

##### Major depressive disorder

SNP-major depressive disorder associations correspond to the discovery stage analysis of Ripke and colleagues under the PGC Major Depressive Disorder Working Group in 9,240 cases and 9,519 controls of European ancestry^45^.

Only 62 BMI-associated SNPs were available. Proxies for missing variants were identified using the SNP Annotation and Proxy Search tool (http://www.broadinstitute.org/mpg/snap/ldsearch.php). A proxy was defined as a genetic variant within 500 kb of the index SNP and in high linkage disequilibrium (*r*^2^ > 0.8) with it. If there was more than one proxy available for the same index SNP, the variant with the higher *r*^2^ was selected. Using 1000 Genomes Pilot 1 (CEU population) as the reference panel, 26 proxies available in both SNP-BMI and SNP-major depressive disorder datasets were identified. An additional search using HapMap release 22 (CEU population) as the reference panel yielded two additional proxies, thus totalizing 28 proxy variants and 90 BMI-associated SNPs. After harmonization, the correlation between effect allele frequencies was 0.989.

##### Schizophrenia

Ripke and colleagues performed SNP-schizophrenia associations under the PGC Schizophrenia Working Group in 34,241 cases and 45,604 ancestry-matched controls (most of European ancestry), and three family-based studies comprising 1,235 parent affected-offspring European ancestry trios^46^. 96 BMI-associated SNPs were available (effect allele frequencies were unavailable).

### Statistical analysis

In Mendelian randomization analyses, all BMI-associated SNPs available for each psychiatric disorder were used (Table 1 and Supplementary Tables S3–S6). The following methods were used:

1. IVW method, consisting of a linear regression of SNP-outcome (dependent variable) on SNP-exposure coefficients (independent variable), weighting by the inverse of the squared SNP-outcome standard errors. The intercept is constrained at zero, which follows from the assumption that SNP-outcome associations are entirely mediated by the exposure^35^. This corresponds to a fixed effects meta-analysis of the ratio estimates from each genetic variant.
2. MR-Egger regression, which differs from the IVW method because the intercept is not constrained. This yields a causal effect estimate robust against horizontal pleiotropy under the InSIDE (Instrument Strength Independent on Direct Effect) assumption, which requires that the SNP-exposure and direct SNP-outcome associations are independent. The intercept provides a test for directional horizontal pleiotropy^36^. Both IVW and MR-Egger regression (as currently implemented) make the so-called NOME (No Measurement Error) assumption. That is, they assume that the SNP-exposure (in this case BMI) association estimate is equal to the true association. NOME violations attenuate the causal effect estimate towards the null in two-sample MR studies, and MR-Egger regression has been shown to be more prone to such attenuation than IVW. Moreover, NOME violations might either inflate or attenuate the MR-Egger intercept (depending on presence of and directional consistency between the intercept and the causal effect estimate). A modified version of the *I^2^* statistic – 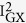 – has been proposed to quantify regression dilution in MR-Egger regression due to NOME violations, which can adjusted for using the SIMEX method^38^.
3. Weighted median method, which provides a valid causal estimate if at least 50% of the weights (ie, the “information” that each genetic instrument contributes to the estimate, which depends on the precision of individual estimates) come from valid instruments, regardless of whether or not horizontal pleiotropic effects of the remaining variants respect the InSIDE assumption^37^.

Point estimates and standard errors were calculated for the IVW, MR-Egger and weighted median methods using the code provided by Bowden *et al*.^36^,^37^. Since SNP-BMI associations were estimated using inverse-transformed BMI, the Mendelian randomization estimates can be interpreted as the odds ratio per 1-SD increment in BMI.

### Sensitivity analyses

Sample overlap between GIANT and PGC datasets can bias causal effect estimates from Mendelian randomization towards the observational (and possibly confounded) estimate^33^,^34^. We evaluated the issue of sample overlap indirectly using a method developed for meta-analysis of dependent “omic” datasets^47^. Briefly, assuming that the null hypothesis is true for most of the genome, correlations between datasets regarding Z-statistics of the SNP-phenotype associations would be expected to be close to zero if there is no sample overlap. To improve robustness against “contamination” due to true signals (ie, common genetic effects), tetrachoric correlations for each pair of BMI-PGC datasets were computed using Z-statistics truncated into two categories: 1 if Z > 0 or 0 if Z ≤ 0.

Mendelian randomization analyses were also performed within SNP subgroups of biological function: neuronal-related (comprising the “neuronal developmental processes”, “neurotransmission”, “hypothalamic expression and regulatory function” and “neuronal expression” categories); and non-neuronal (comprising the remaining categories). Consistency among different biological subgroups would argue against the role of horizontal pleiotropy in the results.

To evaluate if the results were substantially driven by a few instruments, the analyses were repeated excluding influential SNPs. A SNP was classified as influential if at least one of two tests of influence (based on studentized residuals and Cook’s distance) yielded a P-value < 0.05. These were calculated separately for IVW or MR-Egger regression, but the same SNPs were classified as influential using either Mendelian randomization method. The null distributions of these tests were: Student’s t-distribution with degrees of freedom equal to the number of SNPs minus 2, for studentized residuals; or the F-statistic with joint degrees of freedom equal to (1, number of SNPs minus 1) (for IVW) or (1, number of SNPs minus 2) (for MR-Egger regression), for Cook’s distance^48^.

In exploratory analysis aimed at identifying factors associated with horizontal pleiotropy, individual-SNP ratio estimates (in this study, per-allele log odds of a psychiatric disorder divided by the correspondent per-allele change in inverse-transformed BMI residuals units) were used to calculate between-instrument heterogeneity, which corresponds to horizontal pleiotropy in the Mendelian randomization context^49^. Standard errors were obtained using the delta method^50^. Random effects meta-regression was used to evaluate how much of between-instrument heterogeneity (and, therefore, horizontal pleiotropy) can be explained by influentiality status and biological categories. For the latter, an additional analysis using a forward selection process was performed to identify categories that explain heterogeneity. Indicators of belonging to a given biological category were added one at a time based on the largest reduction in *τ^2^*, until no additional covariates reached *P* < 5%.

Two additional sensitivity analyses were performed: (i) IVW estimates within subgroups of SNPs excluding one biological category at a time; (ii) estimation of causal effects using the three Mendelian randomization methods after removing SNPs belonging to the biological categories identified in the forward selection process described above.

Analyses were performed using R 3.2.4 (www.r-project.org).

## Additional Information

**How to cite this article**: Hartwig, F. P. *et al*. Body mass index and psychiatric disorders: a Mendelian randomization study. *Sci. Rep.* **6**, 32730; doi: 10.1038/srep32730 (2016).

## Supplementary Material

Supplementary Information

## Figures and Tables

**Table 1:** Characteristics of each psychiatric disorder dataset.

Characteristic	Bipolar disorder	Schizophrenia	Major depressive disorder^a^
Number of cases/controls	7,481/9,250	34,241/45,604^b^	9,240/9,519
Number of BMI SNPs available	97	96	90
SNP-BMI F-statistic in GIANT			
Mean (standard deviation)	56.54 (75.98)	56.71 (76.36)	54.71 (77.02)
Median (interquartile range)	34.98 (20.08)	34.72 (20.91)	33.34 (24.18)
SNP-BMI F-statistic in PGC^c^			
Mean (standard deviation)	3.01 (3.96)	14.35 (19.00)	3.36 (4.55)
Median (interquartile range)	1.91 (1.25)	9.03 (5.82)	2.00 (1.39)
Overlap between GIANT and each PGC dataset			
Number of SNPs^d^	2,421,360	2,480,664	1,093,724
Tetrachoric correlation^e^	−0.020	−0.026	−0.004

BMI: Body mass index. GIANT: Genetic Investigation of Anthropometric Traits consortium. PGC: Psychiatric Genomics Consortium.

^a^Refers to 62 BMI-associated SNPs reported by the GIANT consortium and 28 proxies.

^b^This refers to the sample size from studies of unrelated individuals only.

^c^These are approximations based on the assumption that SNP-BMI associations are similar in cases and controls and between GIANT and PGC.

^d^Number of SNPs available and that had the same allele pair in both the GIANT and PGC.

^e^Computed using truncated Z-statistics (1 if Z > 0 or 0 if Z ≤ 0) of the SNP-phenotype associations (see Materials and Methods for details).

**Table 2:** Odds ratio (OR) estimates of bipolar disorder, major depressive disorder (MDD) and schizophrenia per 1-standard deviation increment in BMI based on IVW, MR-Egger and weighted median approaches.

Included SNPs^a^	Parameter	Statistic	IVW	Weighted median	MR-Egger	MR-Egger (SIMEX)^b^
**Outcome: Bipolar disorder**						
All	Intercept	Odds (95% CI)	—	—	0.99 (0.97; 1.01)	0.99 (0.97; 1.01)
(97 SNPs)		*P*	—	—	0.281	0.294
	OR	OR (95% CI)	0.90 (0.69; 1.16)	0.88 (0.62; 1.25)	1.23 (0.65; 2.31)	1.26 (0.63; 2.52)
		*P*	0.416	0.482	0.517	0.516
Non-influential	Intercept	Odds (95% CI)	—	—	0.99 (0.98; 1.01)	0.99 (0.98; 1.01)
(92 SNPs)		*P*	—	—	0.319	0.335
	OR	OR (95% CI)	0.93 (0.74; 1.17)	0.88 (0.62; 1.26)	1.19 (0.69; 2.05)	1.21 (0.67; 2.20)
		*P*	0.532	0.487	0.518	0.526
**Outcome: Schizophrenia**						
All	Intercept	Odds (95% CI)	—	—	0.99 (0.98; 1.00)	0.99 (0.97; 1.00)
(96 SNPs)		*P*	—	—	0.101	0.103
	OR	OR (95% CI)	0.98 (0.80; 1.19)	0.93 (0.78; 1.11)	1.41 (0.87; 2.27)	1.46 (0.86; 2.47)
		*P*	0.806	0.420	0.162	0.161
Non-influential	Intercept	Odds (95% CI)	—	—	0.99 (0.98; 1.00)	0.99 (0.98; 1.01)
(92 SNPs)		*P*	—	—	0.280	0.283
	OR	OR (95% CI)	1.01 (0.86; 1.18)	0.93 (0.78; 1.10)	1.22 (0.83; 1.81)	1.25 (0.81; 1.92)
		*P*	0.944	0.406	0.310	0.315
**Outcome: Major depressive disorder**						
All	Intercept	Odds (95% CI)	—	—	1.00 (0.98; 1.01)	1.00 (0.98; 1.01)
(90 SNPs)		*P*	—	—	0.669	0.622
	OR	OR (95% CI)	1.15 (0.92; 1.44)	1.40 (1.03; 1.90)	1.28 (0.74; 2.24)	1.33 (0.72; 2.47)
		*P*	0.221	0.035	0.374	0.364
Non-influential	Intercept	Odds (95% CI)	—	—	0.99 (0.98; 1.01)	0.99 (0.98; 1.01)
(86 SNPs)		*P*	—	—	0.385	0.328
	OR	OR (95% CI)	1.25 (1.02; 1.52)	1.45 (1.05; 1.99)	1.52 (0.93; 2.49)	1.60 (0.93; 2.75)
		*P*	0.030	0.026	0.094	0.087

95% CI: 95% Confidence interval. *P*: P-value. SIMEX: Simulation Extrapolation.

^a^All corresponds to Mendelian randomization analysis using all BMI-associated SNPs available in the correspondent psychiatric dataset. “Non-influential” is similar, but excludes SNPs classified as influential using statistical tests based on studentized residuals and Cook’s distance (see Materials and Methods for details).

^b^This differs from regular MR-Egger regression because it uses the SIMEX method to correct for regression dilution bias.

**Table 3:** Odds ratio (OR) estimates of bipolar disorder, major depressive disorder and schizophrenia per 1- standard deviation increment in BMI based on IVW, MR-Egger and weighted median approaches, within independent subgroups of SNPs defined using biological criteria.

Influential SNPs	SNP subgroup	Statistic	IVW	Weighted median	MR-Egger	MR-Egger (SIMEX)^e^
**Outcome: Bipolar disorder**						
Included^a^	Neuronal^c^	OR (95% CI)	0.93 (0.66; 1.32)	0.89 (0.59; 1.34)	1.19 (0.54; 2.60)	1.19 (0.51; 2.75)
	(54 SNPs)	*P*	0.684	0.570	0.662	0.680
	Other^d^	OR (95% CI)	0.83 (0.55; 1.27)	0.97 (0.57; 1.64)	1.33 (0.38; 4.68)	1.42 (0.32; 6.34)
	(43 SNPs)	*P*	0.386	0.902	0.645	0.636
Excluded^b^	Neuronal^c^	OR (95% CI)	0.94 (0.70; 1.27)	0.89 (0.59; 1.34)	1.09 (0.56; 2.10)	1.09 (0.54; 2.20)
	(50 SNPs)	*P*	0.698	0.574	0.802	0.809
	Other^d^	OR (95% CI)	0.90 (0.62; 1.32)	0.97 (0.57; 1.66)	1.62 (0.52; 5.02)	1.81 (0.47; 6.97)
	(42 SNPs)	*P*	0.597	0.917	0.394	0.377
**Outcome: Schizophrenia**						
Included^a^	Neuronal^c^	OR (95% CI)	1.00 (0.80; 1.26)	0.93 (0.76; 1.13)	1.30 (0.77; 2.21)	1.33 (0.75; 2.34)
	(54 SNPs)	*P*	0.992	0.474	0.317	0.318
	Other^d^	OR (95% CI)	0.93 (0.64; 1.35)	0.86 (0.66; 1.13)	1.75 (0.58; 5.24)	1.95 (0.53; 7.15)
	(42 SNPs)	*P*	0.687	0.285	0.311	0.306
Excluded^b^	Neuronal^c^	OR (95% CI)	1.06 (0.86; 1.30)	0.93 (0.76; 1.13)	1.15 (0.71; 1.86)	1.17 (0.70; 1.95)
	(52 SNPs)	*P*	0.602	0.472	0.554	0.545
	Other^d^	OR (95% CI)	0.91 (0.70; 1.20)	0.86 (0.66; 1.12)	1.37 (0.61; 3.09)	1.46 (0.55; 3.88)
	(40 SNPs)	*P*	0.495	0.271	0.442	0.440
**Outcome: Major depressive disorder**						
Included^a^	Neuronal^c^	OR (95% CI)	1.16 (0.90; 1.50)	1.44 (1.01; 2.06)	1.26 (0.70; 2.27)	1.29 (0.69; 2.43)
	(54 SNPs)	*P*	0.254	0.051	0.424	0.422
	Other^d^	OR (95% CI)	1.13 (0.71; 1.80)	1.36 (0.81; 2.28)	1.38 (0.28; 6.70)	1.52 (0.21; 10.83)
	(36 SNPs)	*P*	0.594	0.257	0.680	0.669
Excluded^b^	Neuronal^c^	OR (95% CI)	1.20 (0.94; 1.53)	1.48 (1.02; 2.15)	1.60 (0.92; 2.77)	1.66 (0.92; 3.00)
	(52 SNPs)	*P*	0.142	0.042	0.096	0.089
	Other^d^	OR (95% CI)	1.37 (0.95; 1.98)	1.39 (0.82; 2.35)	1.46 (0.43; 4.97)	1.62 (0.36; 7.32)
	(34 SNPs)	*P*	0.090	0.227	0.536	0.517

95% CI: 95% Confidence interval. *P*: P-value. SIMEX: Simulation Extrapolation.

^a^SNPs classified as influential using statistical tests based on studentized residuals and Cook’s distance (see Materials and Methods for details) were included.

^b^SNPs classified as influential were excluded.

^c^Includes SNPs belonging to “neuronal developmental processes”, “neurotransmission”, “hypothalamic expression and regulatory function” or “neuronal Expression” biological categories.

^d^Includes SNPs belonging to the remaining biological categories.

^e^This differs from regular MR-Egger regression because it uses the SIMEX method to correct for regression dilution bias.

**Table 4:** Odds ratio (OR) estimates of bipolar disorder, major depressive disorder (MDD) and schizophrenia per 1- standard deviation increment in BMI based on the IVW approach, within subgroups of SNPs excluding one biological category at a time.

Excluded biological category	Bipolar disorder			Schizophrenia			MDD		
	Number of SNPs	OR (95% CI)	*P*	Number of SNPs	OR (95% CI)	*P*	Number of SNPs	OR (95% CI)	*P*
Neuronal development^a^	68	0.88 (0.66; 1.18)	0.383	67	0.97 (0.77; 1.22)	0.792	61	1.13 (0.85; 1.51)	0.382
Neurotransmission	87	0.95 (0.72; 1.25)	0.718	86	1.04 (0.85; 1.28)	0.695	80	1.16 (0.91; 1.49)	0.228
Hypothalamus-related^a^	84	0.75 (0.55; 1.03)	0.077	83	0.92 (0.71; 1.18)	0.484	77	1.07 (0.82; 1.41)	0.606
Neuronal expression	85	0.92 (0.70; 1.21)	0.552	84	0.99 (0.80; 1.22)	0.932	78	1.15 (0.90; 1.47)	0.250
Lipid-related^a^	87	0.95 (0.73; 1.23)	0.674	86	1.04 (0.86; 1.26)	0.680	80	1.25 (1.00; 1.56)	0.051
Bone development	88	0.93 (0.71; 1.22)	0.588	87	0.97 (0.78; 1.20)	0.750	82	1.19 (0.94; 1.51)	0.149
MAPK1/Extracellular kinases^a^	88	0.92 (0.71; 1.21)	0.559	87	0.97 (0.79; 1.19)	0.777	81	1.16 (0.92; 1.46)	0.217
Endocytosis/Exocytosis	83	0.98 (0.75; 1.28)	0.903	82	1.01 (0.81; 1.25)	0.963	76	1.12 (0.87; 1.44)	0.368
Tumorigenesis	86	0.92 (0.70; 1.20)	0.542	85	1.02 (0.82; 1.25)	0.885	79	1.16 (0.91; 1.48)	0.235
Apoptosis	84	0.92 (0.71; 1.21)	0.555	83	0.98 (0.79; 1.21)	0.837	78	1.23 (0.98; 1.54)	0.078
Membrane proteins	86	0.91 (0.68; 1.22)	0.534	85	0.98 (0.79; 1.23)	0.876	79	1.23 (0.96; 1.58)	0.097
Monogenic obesity/Energy^a^	88	0.85 (0.63; 1.14)	0.271	87	0.94 (0.75; 1.18)	0.588	81	1.17 (0.90; 1.51)	0.229
Immune system	82	0.92 (0.70; 1.20)	0.527	81	0.98 (0.78; 1.22)	0.853	77	1.19 (0.93; 1.52)	0.165
Glucose homeostasis/diabetes^a^	86	0.90 (0.68; 1.19)	0.448	85	0.97 (0.78; 1.19)	0.743	79	1.29 (1.01; 1.64)	0.038
Cell cycle	74	0.95 (0.71; 1.26)	0.715	73	1.00 (0.79; 1.27)	0.985	70	1.06 (0.81; 1.39)	0.644
Unspecified^a^	72	1.06 (0.81; 1.41)	0.655	72	1.03 (0.83; 1.28)	0.768	66	1.26 (0.97; 1.62)	0.081

95% CI: 95% Confidence interval. *P*: P-value.

^a^**Neuronal development:** Neuronal developmental processes**. Hypothalamus-related:** Hypothalamic expression and regulatory function. **Lipid-related:** Lipid biosynthesis and metabolism. **MAPK1/Extracellular kinases:** Mitogen activated protein kinase 1/Extracellular signal-regulated kinases. **Monogenic obesity/Energy:** Monogenic Obesity and/or Energy Homeostasis. **Glucose homeostasis/diabetes:** Glucose homeostasis and/or diabetes. **Unspecified:** Prioritized by GRAIL-Putative coding variant annotation-CNV-eQTL-DEPICT but not in above categories.

## References

[b1] Swinburn B. A. . The global obesity pandemic: shaped by global drivers and local environments. Lancet 378, 804–814 (2011). 21872749 10.1016/S0140-6736(11)60813-1

[b2] Global Burden of Disease Study 2013 Collaborators. Global, regional, and national incidence, prevalence, and years lived with disability for 301 acute and chronic diseases and injuries in 188 countries, 1990–2013: a systematic analysis for the Global Burden of Disease Study 2013. Lancet 386, 743–800 (2015). 26063472 10.1016/S0140-6736(15)60692-4 PMC4561509

[b3] Liem E. T., Sauer P. J., Oldehinkel A. J. & Stolk R. P. Association between depressive symptoms in childhood and adolescence and overweight in later life: review of the recent literature. Arch Pediatr Adolesc Med 162, 981–988 (2008). 18838652 10.1001/archpedi.162.10.981

[b4] Abou Abbas L., Salameh P., Nasser W., Nasser Z. & Godin I. Obesity and symptoms of depression among adults in selected countries of the Middle East: a systematic review and meta-analysis. Clin Obes 5, 2–11 (2015). 25504829 10.1111/cob.12082

[b5] Baskaran A., Cha D. S., Powell A. M., Jalil D. & McIntyre R. S. Sex differences in rates of obesity in bipolar disorder: postulated mechanisms. Bipolar Disord 16, 83–92 (2014). 24467470 10.1111/bdi.12141

[b6] McElroy S. L. & Keck P. E. Jr. Obesity in bipolar disorder: an overview. Curr Psychiatry Rep 14, 650–658 (2012). 22903246 10.1007/s11920-012-0313-8

[b7] Limosin F., Gasquet I., Leguay D., Azorin J. M. & Rouillon F. Body mass index and prevalence of obesity in a French cohort of patients with schizophrenia. Acta Psychiatr Scand 118, 19–25 (2008). 18582344 10.1111/j.1600-0447.2008.01208.x

[b8] Saarni S. E. . Body composition in psychotic disorders: a general population survey. Psychol Med 39, 801–810 (2009). 18713488 10.1017/S0033291708004194

[b9] Citrome L. & Vreeland B. Schizophrenia, obesity, and antipsychotic medications: what can we do? Postgrad Med 120, 18–33 (2008). 18654065 10.3810/pgm.2008.07.1786

[b10] Wildes J. E., Marcus M. D. & Fagiolini A. Obesity in patients with bipolar disorder: a biopsychosocial-behavioral model. J Clin Psychiatry 67, 904–915 (2006). 16848650 10.4088/jcp.v67n0607

[b11] Vancampfort D. . Relationships between obesity, functional exercise capacity, physical activity participation and physical self-perception in people with schizophrenia. Acta Psychiatr Scand 123, 423–430 (2011). 21219266 10.1111/j.1600-0447.2010.01666.x

[b12] Luppino F. S. . Overweight, obesity, and depression: a systematic review and meta-analysis of longitudinal studies. Arch Gen Psychiatry 67, 220–229 (2010). 20194822 10.1001/archgenpsychiatry.2010.2

[b13] Daly M. The relationship of C-reactive protein to obesity-related depressive symptoms: a longitudinal study. Obesity (*Silver Spring*) 21, 248–250 (2013). 23404927 10.1002/oby.20051

[b14] Liu C. S., Carvalho A. F., Mansur R. B. & McIntyre R. S. Obesity and bipolar disorder: synergistic neurotoxic effects? Adv Ther 30, 987–1006 (2013). 24194362 10.1007/s12325-013-0067-7

[b15] Thakore J. H., Mann J. N., Vlahos I., Martin A. & Reznek R. Increased visceral fat distribution in drug-naive and drug-free patients with schizophrenia. Int J Obes Relat Metab Disord 26, 137–141 (2002). 11791159 10.1038/sj.ijo.0801840

[b16] Ryan M. C., Flanagan S., Kinsella U., Keeling F. & Thakore J. H. The effects of atypical antipsychotics on visceral fat distribution in first episode, drug-naive patients with schizophrenia. Life Sci 74, 1999–2008 (2004). 14967195 10.1016/j.lfs.2003.08.044

[b17] Sengupta S. . Are metabolic indices different between drug-naive first-episode psychosis patients and healthy controls? Schizophr Res 102, 329–336 (2008). 18396386 10.1016/j.schres.2008.02.013

[b18] Padmavati R., McCreadie R. G. & Tirupati S. Low prevalence of obesity and metabolic syndrome in never-treated chronic schizophrenia. Schizophr Res 121, 199–202 (2010). 20538429 10.1016/j.schres.2010.05.010

[b19] Bjorngaard J. H. . Association of Body Mass Index with Depression, Anxiety and Suicide-An Instrumental Variable Analysis of the HUNT Study. PLoS One 10, e0131708 (2015). 26167892 10.1371/journal.pone.0131708 PMC4500562

[b20] Phillips A. N. & Davey Smith G. How independent are “independent” effects? Relative risk estimation when correlated exposures are measured imprecisely. J Clin Epidemiol 44, 1223–1231 (1991). 1941017 10.1016/0895-4356(91)90155-3

[b21] Davey Smith G. Use of genetic markers and gene-diet interactions for interrogating population-level causal influences of diet on health. Genes Nutr 6, 27–43 (2011). 21437028 10.1007/s12263-010-0181-y PMC3040803

[b22] Davey Smith G. . Clustered environments and randomized genes: a fundamental distinction between conventional and genetic epidemiology. PLoS Med 4, e352 (2007). 18076282 10.1371/journal.pmed.0040352 PMC2121108

[b23] Davey Smith G. & Ebrahim S. ‘Mendelian randomization’: can genetic epidemiology contribute to understanding environmental determinants of disease? Int J Epidemiol 32, 1–22 (2003). 12689998 10.1093/ije/dyg070

[b24] Locke A. E. . Genetic studies of body mass index yield new insights for obesity biology. Nature 518, 197–206 (2015). 25673413 10.1038/nature14177 PMC4382211

[b25] Kivimaki M. . Examining overweight and obesity as risk factors for common mental disorders using fat mass and obesity-associated (FTO) genotype-instrumented analysis: The Whitehall II Study, 1985–2004. Am J Epidemiol 173, 421–429 (2011). 21248310 10.1093/aje/kwq444 PMC3032807

[b26] Lawlor D. A. . Using genetic loci to understand the relationship between adiposity and psychological distress: a Mendelian Randomization study in the Copenhagen General Population Study of 53,221 adults. J Intern Med 269, 525–537 (2011). 21210875 10.1111/j.1365-2796.2011.02343.x

[b27] Cronin R. M. . Phenome-wide association studies demonstrating pleiotropy of genetic variants within FTO with and without adjustment for body mass index. Front Genet 5, 250 (2014). 25177340 10.3389/fgene.2014.00250 PMC4134007

[b28] Huang H. & Tao Y. X. Pleiotropic functions of the transmembrane domain 6 of human melanocortin-4 receptor. J Mol Endocrinol 49, 237–248 (2012). 23014839 10.1530/JME-12-0161

[b29] Walter S. . Revisiting Mendelian randomization studies of the effect of body mass index on depression. Am J Med Genet B Neuropsychiatr Genet 168B, 108–115 (2015). 25656382 10.1002/ajmg.b.32286 PMC4387873

[b30] Glymour M. M., Tchetgen Tchetgen E. J. & Robins J. M. Credible Mendelian randomization studies: approaches for evaluating the instrumental variable assumptions. Am J Epidemiol 175, 332–339 (2012). 22247045 10.1093/aje/kwr323 PMC3366596

[b31] Jokela M. . Body mass index and depressive symptoms: instrumental-variables regression with genetic risk score. Genes, brain, and behavior 11, 942–948 (2012). 10.1111/j.1601-183X.2012.00846.x 22958333

[b32] Hung C. F. . Relationship between obesity and the risk of clinically significant depression: Mendelian randomisation study. The British journal of psychiatry: the journal of mental science 205, 24–28 (2014). 24809401 10.1192/bjp.bp.113.130419 PMC4076654

[b33] Pierce B. L. & Burgess S. Efficient design for Mendelian randomization studies: subsample and 2-sample instrumental variable estimators. Am J Epidemiol 178, 1177–1184 (2013). 23863760 10.1093/aje/kwt084 PMC3783091

[b34] Hartwig F. P. & Davies N. M. Why internal weights should be avoided (not only) in MR-Egger regression. Int J Epidemiol In press (2016). 10.1093/ije/dyw240 27649799

[b35] Burgess S., Butterworth A. & Thompson S. G. Mendelian randomization analysis with multiple genetic variants using summarized data. Genet Epidemiol 37, 658–665 (2013). 24114802 10.1002/gepi.21758 PMC4377079

[b36] Bowden J., Davey Smith G. & Burgess S. Mendelian randomization with invalid instruments: effect estimation and bias detection through Egger regression. Int J Epidemiol 44, 512–525 (2015). 26050253 10.1093/ije/dyv080 PMC4469799

[b37] Bowden J., Davey Smith G., Haycock P. C. & Burgess S. Consistent Estimation in Mendelian Randomization with Some Invalid Instruments Using a Weighted Median Estimator. Genet Epidemiol 40, 304–314 (2016). 27061298 10.1002/gepi.21965 PMC4849733

[b38] Bowden J. . Asessing the validity of MR-Egger regression for two-sample Mendelian randomization: the role of the *I*^2^ statistic. Int J Epidemiol In press (2016). 10.1093/ije/dyw220 PMC5446088 27616674

[b39] Martin J. . Association of Genetic Risk for Schizophrenia With Nonparticipation Over Time in a Population-Based Cohort Study. Am J Epidemiol 183, 1149–1158 (2016). 27188935 10.1093/aje/kww009 PMC4908211

[b40] Lopresti A. L. & Drummond P. D. Obesity and psychiatric disorders: commonalities in dysregulated biological pathways and their implications for treatment. Prog Neuropsychopharmacol Biol Psychiatry 45, 92–99 (2013). 23685202 10.1016/j.pnpbp.2013.05.005

[b41] Khandaker G. M. . Inflammation and immunity in schizophrenia: implications for pathophysiology and treatment. Lancet Psychiatry 2, 258–270 (2015). 26359903 10.1016/S2215-0366(14)00122-9 PMC4595998

[b42] Wium-Andersen M. K., Orsted D. D. & Nordestgaard B. G. Elevated C-reactive protein, depression, somatic diseases, and all-cause mortality: a mendelian randomization study. Biol Psychiatry 76, 249–257 (2014). 24246360 10.1016/j.biopsych.2013.10.009

[b43] Herman M. A. & Rosen E. D. Making Biological Sense of GWAS Data: Lessons from the FTO Locus. Cell Metab 22, 538–539 (2015). 26445508 10.1016/j.cmet.2015.09.018

[b44] Psychiatric G. W. A. S. Consortium Bipolar Disorder Working Group. Large-scale genome-wide association analysis of bipolar disorder identifies a new susceptibility locus near ODZ4. Nat Genet 43, 977–983 (2011). 21926972 10.1038/ng.943 PMC3637176

[b45] Ripke S. . A mega-analysis of genome-wide association studies for major depressive disorder. Mol Psychiatry 18, 497–511 (2013). 22472876 10.1038/mp.2012.21 PMC3837431

[b46] Schizophrenia Working Group of the Psychiatric Genomics Consortium. Biological insights from 108 schizophrenia-associated genetic loci. Nature 511, 421–427 (2014). 25056061 10.1038/nature13595 PMC4112379

[b47] Province M. A. & Borecki I. B. A correlated meta-analysis strategy for data mining “OMIC” scans. Pac Symp Biocomput, 236–246 (2013). 23424128 PMC3773990

[b48] Corbin L. J. . Body mass index as a modifiable risk factor for type 2 diabetes: Refining and understanding causal estimates using Mendelian randomisation. Diabetes (2016). 10.2337/db16-0418 PMC5279886 27402723

[b49] Greco M. F., Minelli C., Sheehan N. A. & Thompson J. R. Detecting pleiotropy in Mendelian randomisation studies with summary data and a continuous outcome. Stat Med 34, 2926–2940 (2015). 25950993 10.1002/sim.6522

[b50] Thomas D. C., Lawlor D. A. & Thompson J. R. Re: Estimation of bias in nongenetic observational studies using “Mendelian triangulation” by Bautista *et al*. Ann Epidemiol 17, 511–513 (2007). 17466535 10.1016/j.annepidem.2006.12.005

