# Supplementary material for: Body mass index and psychiatric disorders: a Mendelian randomization study
Source: Sci Rep. 2016 Sep 7;6:32730. doi: 10.1038/srep32730 (PMC5013405; doi:10.1038/srep32730)
Supplement: Supplementary Information [file srep32730-s1.pdf]

Body mass index and psychiatric disorders: a Mendelian randomization study

Fernando Pires Hartwig[1]*, Jack Bowden[2], Christian Loret de Mola[1], Luciana Tovo-Rodrigues[1], George Davey Smith[2] and Bernardo Lessa Horta[1]

[1]Postgraduate Program in Epidemiology, Federal University of Pelotas, Pelotas, Brazil.

[2]MRC Integrative Epidemiology Unit, University of Bristol, Bristol, UK

*Corresponding author. Postgraduate Program in Epidemiology, Federal University of Pelotas, Pelotas (Brazil) 96020-220. Phone: 55 53 81347172. E-mail: fernandophartwig@gmail.com.

SUPPLEMENTARY TABLES

Supplementary Table S1. Heterogeneity statistics from random effects meta-regression of individual-SNP log odds ratio of bipolar disorder, major depressive disorder (MDD) and schizophrenia per 1-standard deviation increment in BMI throughout a forward selection process of potential biological moderators.

Selection step[a]	Biological category included	τ^2	I^2		Adjusted R^2	
			%	P-value	%	P-value
**Outcome: Bipolar disorder**						
0	None	0.45	29.1	0.005	-	-
1	Unspecified[c]	0.35	24.1	0.021	22.5	0.003
2[b]	Endocytosis / Exocytosis	0.30	21.5	0.038	32.9	0.024
3	Hypothalamus-related[c]	0.32	22.0	0.035	28.5	0.261
**Outcome: Schizophrenia**						
0	None	0.49	68.8	9.9×10^{-24}	-	-
1	Neurotransmission	0.47	67.5	1.0×10^{-21}	5.0	0.008
2[b]	Lipid-related[c]	0.45	66.7	1.5×10^{-20}	8.3	0.024
3	Tumorigenesis	0.46	66.8	1.9×10^{-20}	7.7	0.161
**Outcome: Major depressive disorder**						
0	None	0.20	18.4	0.073	-	-
1	Lipid-related[c]	0.13	12.9	0.161	34.1	0.007
2[b]	Glucose homeostasis / diabetes[c]	0.06	6.5	0.309	68.8	0.012
3	Bone development	0.04	4.1	0.371	80.5	0.078

[a]In each step of the forward selection process, the results for the included moderator are shown adjusting for all moderators included in previous steps.
[b]The last moderator that met the inclusion criterion of $P<0.05$.
[c]**Unspecified:** Prioritized by GRAIL-Putative coding variant annotation-CNV-eQTL-DEPICT but not in above categories. **Hypothalamus-related:** Hypothalamic expression and regulatory function. **Lipid-related:** Lipid biosynthesis and metabolism. **Lipid-related:** Lipid biosynthesis and metabolism.

Supplementary Table S2. Odds ratio (OR) estimates of bipolar disorder, major depressive disorder (MDD) and schizophrenia per 1- standard deviation increment in BMI based on IVW, MR-Egger and weighted median approaches after excluding SNPs belonging to selected biological categories.

**Excluded biological categories (Number of SNPs)**	**Statistic**	**IVW**	**Weighted median**	**MR-Egger**	**MR-Egger (SIMEX)**[e]
**Outcome: Bipolar disorder**					
Unspecified[a]	OR (95% CI)	1.06 (0.81; 1.41)	0.89 (0.62; 1.27)	1.20 (0.62; 2.32)	1.22 (0.60; 2.49)
(72)	*P*	0.655	0.513	0.581	0.580
Endocytosis / Exocytosis	OR (95% CI)	0.98 (0.75; 1.28)	0.88 (0.62; 1.26)	1.02 (0.54; 1.94)	1.03 (0.51; 2.08)
(83)	*P*	0.903	0.493	0.945	0.931
Both[b]	OR (95% CI)	1.14 (0.86; 1.53)	0.98 (0.66; 1.43)	1.06 (0.55; 2.07)	1.07 (0.52; 2.20)
(62)	*P*	0.356	0.901	0.859	0.850
**Outcome: Schizophrenia**					
Neurotransmission	OR (95% CI)	1.04 (0.85; 1.28)	0.96 (0.81; 1.14)	1.37 (0.84; 2.24)	1.41 (0.82; 2.44)
(86)	*P*	0.695	0.635	0.210	0.215
Lipid-related[a]	OR (95% CI)	1.04 (0.86; 1.26)	0.93 (0.78; 1.11)	1.41 (0.89; 2.22)	1.46 (0.88; 2.41)
(86)	*P*	0.680	0.419	0.138	0.137
Both[c]	OR (95% CI)	1.10 (0.90; 1.35)	1.10 (0.92; 1.30)	1.43 (0.89; 2.31)	1.50 (0.89; 2.52)
(77)	*P*	0.338	0.309	0.135	0.127
**Outcome: Major depressive disorder**					
Lipid-related[a]	OR (95% CI)	1.25 (1.00; 1.56)	1.43 (1.03; 1.98)	1.19 (0.70; 2.04)	1.21 (0.67; 2.19)
(80)	*P*	0.051	0.035	0.516	0.521
Glucose homeostasis / diabetes[a]	OR (95% CI)	1.29 (1.01; 1.64)	1.52 (1.08; 2.12)	1.52 (0.84; 2.76)	1.60 (0.83; 3.11)
(79)	*P*	0.038	0.017	0.163	0.160
Both[d]	OR (95% CI)	1.38 (1.09; 1.75)	1.55 (1.10; 2.19)	1.43 (0.80; 2.53)	1.49 (0.78; 2.81)
(71)	*P*	0.008	0.016	0.223	0.221

95% CI: 95% Confidence interval. *P*: P-value. SIMEX: Simulation Extrapolation.
[a]**Unspecified:** Prioritized by GRAIL-Putative coding variant annotation-CNV-eQTL-DEPICT but not in above categories. **Hypothalamus-related:** Hypothalamic expression and regulatory function. **Lipid-related:** Lipid biosynthesis and metabolism. **Lipid-related:** Lipid biosynthesis and metabolism.
[b]Excluded categories: Unspecified and Endocytosis / Exocytosis.
[c]Excluded categories: Neurotransmission and Lipid-related.
[d]Excluded categories: Lipid-related and Glucose homeostasis / diabetes.
[e]This differs from regular MR-Egger regression because it uses the SIMEX method to correct for regression dilution bias.

Supplementary Table S3. SNP-BMI regression coefficients corresponding to per-allele standard deviation changes in BMI changes in (β) and associated standard errors (SE) for 125 BMI-associated SNPs (97 reported by the GIANT consortium and 28 proxies for missing variants in the major depressive disorder dataset).

SNP	Proxy	Gene locus[a]	Effect allele	Other allele	Effect allele frequency[b]	β	SE
rs1000940	No	RABEP1	G	A	0.2250	0.0192	0.0034
rs10132280	No	STXBP6	A	C	0.3333	-0.0230	0.0034
rs1016287	No	LINC01122	T	C	0.3250	0.0229	0.0034
rs10182181	No	ADCY3	A	G	0.5000	-0.0307	0.0031
rs10733682	No	LMX1B	A	G	0.4250	0.0174	0.0031
rs10938397	No	GNPDA2	A	G	0.5667	-0.0402	0.0031
rs10968576	No	LINGO2	G	A	0.2917	0.0249	0.0033
rs11030104	No	BDNF	A	G	0.8000	0.0414	0.0038
rs11057405	No	CLIP1	A	G	0.0917	-0.0307	0.0055
rs11126666	No	KCNK3	G	A	0.6917	-0.0207	0.0034
rs11165643	No	PTBP2	C	T	0.4250	-0.0218	0.0031
rs11191560	No	NT5C2	T	C	0.9417	-0.0308	0.0053
rs11583200	No	ELAVL4	C	T	0.3750	0.0177	0.0031
rs1167827	No	HIP1	A	G	0.4583	-0.0202	0.0033
rs11688816	No	EHBP1	A	G	0.5417	-0.0172	0.0031
rs11727676	No	HHIP	C	T	0.0750	-0.0358	0.0064
rs11847697	No	PRKD1	T	C	0.0417	0.0492	0.0084
rs12016871	No	MTIF3	C	T	0.7667	-0.0298	0.0047
rs12286929	No	CADM1	G	A	0.4333	0.0217	0.0031
rs12401738	No	FUBP1	A	G	0.4250	0.0211	0.0033
rs12429545	No	OLFM4	G	A	0.9000	-0.0334	0.0047
rs12446632	No	GPRC5B	A	G	0.1333	-0.0403	0.0046
rs12566985	No	FPGT-TNNI3K	G	A	0.4250	0.0242	0.0031
rs12885454	No	PRKD1	C	A	0.6333	0.0207	0.0033
rs12940622	No	RPTOR	A	G	0.4583	-0.0182	0.0031
rs13021737	No	TMEM18	A	G	0.1250	-0.0601	0.0040
rs13078960	No	CADM2	T	G	0.8167	-0.0297	0.0039
rs13107325	No	SLC39A8	C	T	0.8833	-0.0477	0.0068
rs13191362	No	PARK2	A	G	0.8000	0.0277	0.0048
rs13201877	No	IFNGR1	A	G	0.9167	-0.0233	0.0045
rs1441264	No	MIR548A2	A	G	0.5500	0.0175	0.0032
rs1460676	No	FIGN	T	C	0.7833	-0.0197	0.0040
rs1516725	No	ETV5	T	C	0.0917	-0.0451	0.0046
rs1528435	No	UBE2E3	T	C	0.5833	0.0178	0.0031
rs1558902	No	FTO	A	T	0.4500	0.0818	0.0031
rs16851483	No	RASA2	G	T	0.9083	-0.0483	0.0077
rs16907751	No	ZBTB10	C	T	0.9583	0.0350	0.0066
rs16951275	No	MAP2K5	C	T	0.2250	-0.0311	0.0037
rs17001654	No	SCARB2	C	G	0.8417	-0.0306	0.0053
rs17024393	No	GNAT2	C	T	0.0417	0.0658	0.0088
rs17094222	No	HIF1AN	C	T	0.2083	0.0249	0.0038

rs17203016	No	CREB1	G	A	0.2000	0.0210	0.0039
rs17405819	No	HNF4G	C	T	0.3667	-0.0224	0.0033
rs17724992	No	PGPEP1	A	G	0.6917	0.0194	0.0035
rs1808579	No	C18orf8	T	C	0.4750	-0.0167	0.0031
rs1928295	No	TLR4	C	T	0.4250	-0.0188	0.0031
rs2033529	No	TDRG1	G	A	0.2583	0.0190	0.0033
rs2033732	No	RALYL	C	T	0.7583	0.0192	0.0035
rs205262	No	C6orf106	A	G	0.7333	-0.0221	0.0035
rs2075650	No	TOMM40	A	G	0.8583	0.0258	0.0045
rs2080454	No	CBLN1	A	C	0.6083	-0.0168	0.0031
rs2112347	No	POC5	G	T	0.3750	-0.0261	0.0031
rs2121279	No	LRP1B	T	C	0.1167	0.0245	0.0044
rs2176040	No	LOC646736	G	A	0.6083	-0.0141	0.0031
rs2176598	No	HSD17B12	T	C	0.2000	0.0198	0.0036
rs2207139	No	TFAP2B	G	A	0.1000	0.0447	0.0040
rs2245368	No	PMS2L11	T	C	0.7583	-0.0317	0.0057
rs2287019	No	QPCTL	C	T	0.8500	0.0360	0.0042
rs2365389	No	FHIT	C	T	0.6583	0.0200	0.0031
rs2650492	No	SBK1	A	G	0.3083	0.0207	0.0035
rs2820292	No	NAV1	A	C	0.4917	-0.0195	0.0031
rs2836754	No	ETS2	C	T	0.6500	0.0164	0.0032
rs29941	No	KCTD15	A	G	0.3333	-0.0182	0.0033
rs3101336	No	NEGR1	T	C	0.3509	-0.0334	0.0031
rs3736485	No	DMXL2	A	G	0.4250	0.0176	0.0031
rs3810291	No	ZC3H4	A	G	0.6250	0.0283	0.0036
rs3817334	No	MTCH2	C	T	0.5500	-0.0262	0.0031
rs3849570	No	GBE1	A	C	0.3667	0.0188	0.0034
rs3888190	No	ATP2A1	A	C	0.3583	0.0309	0.0031
rs4256980	No	TRIM66	G	C	0.7250	0.0209	0.0031
rs4740619	No	C9orf93	T	C	0.5333	0.0179	0.0031
rs4787491	No	INO80E	A	G	0.3860	-0.0159	0.0034
rs492400	No	USP37	T	C	0.6750	-0.0158	0.0031
rs543874	No	SEC16B	G	A	0.2667	0.0482	0.0039
rs6091540	No	ZFP64	C	T	0.7250	0.0188	0.0035
rs6465468	No	ASB4	G	T	0.6750	-0.0166	0.0035
rs6477694	No	EPB41L4B	C	T	0.3583	0.0174	0.0031
rs6567160	No	MC4R	C	T	0.2833	0.0556	0.0036
rs657452	No	AGBL4	A	G	0.4167	0.0227	0.0031
rs6804842	No	RARB	A	G	0.4250	-0.0185	0.0031
rs7138803	No	BCDIN3D	G	A	0.5583	-0.0315	0.0031
rs7141420	No	NRXN3	T	C	0.6167	0.0235	0.0031
rs7164727	No	LOC100287559	T	C	0.7750	0.0180	0.0033
rs7239883	No	LOC284260	G	A	0.3167	0.0164	0.0031
rs7243357	No	GRP	G	T	0.1333	-0.0217	0.0040
rs758747	No	NLRC3	C	T	0.7333	-0.0225	0.0037
rs7599312	No	ERBB4	G	A	0.7083	0.0220	0.0034
rs7715256	No	GALNT10	G	T	0.4500	0.0163	0.0031
rs7899106	No	GRID1	A	G	0.9500	-0.0395	0.0071
rs7903146	No	TCF7L2	T	C	0.2500	-0.0234	0.0034
rs9374842	No	LOC285762	T	C	0.7417	0.0187	0.0035
rs9400239	No	FOXO3	C	T	0.7000	0.0188	0.0033
rs9540493	No	MIR548X2	G	A	0.5500	-0.0172	0.0033

rs9641123	No	CALCR	G	C	0.6083	-0.0191	0.0038
rs977747	No	TAL1	T	G	0.4667	0.0167	0.0031
rs9914578	No	SMG6	G	C	0.1667	0.0201	0.0038
rs9925964	No	KAT8	G	A	0.3917	-0.0192	0.0031
rs10464483	Yes	CALCR	C	T	0.6250	-0.0183	0.0038
rs10513801	Yes	ETV5	G	T	0.0917	-0.0448	0.0047
rs10808859	Yes	RALYL	A	T	0.7667	0.0210	0.0044
rs10838184	Yes	HSD17B12	C	G	0.2000	0.0205	0.0043
rs10840100	Yes	TRIM66	G	A	0.7250	0.0209	0.0031
rs11625899	Yes	PRKD1	T	C	0.3667	-0.0199	0.0038
rs11672660	Yes	QPCTL	C	T	0.8250	0.0345	0.0040
rs12369179	Yes	CLIP1	T	C	0.0750	-0.0306	0.0074
rs12641981	Yes	GNPDA2	C	T	0.5667	-0.0399	0.0031
rs13012571	Yes	TMEM18	C	T	0.1250	-0.0597	0.0049
rs13098327	Yes	CADM2	G	A	0.8167	-0.0295	0.0039
rs1421085	Yes	FTO	C	T	0.4500	0.0813	0.0031
rs1462433	Yes	HNF4G	A	G	0.3667	-0.0257	0.0041
rs1515104	Yes	LOC646736	A	T	0.6486	-0.0169	0.0054
rs1885988	Yes	MTIF3	T	C	0.7833	-0.0221	0.0039
rs1978487	Yes	KAT8	C	T	0.3917	-0.0184	0.0038
rs2241420	Yes	MAP2K5	A	G	0.2333	-0.0293	0.0038
rs3797580	Yes	POC5	G	A	0.3583	-0.0247	0.0031
rs3904532	Yes	IFNGR1	A	G	0.9167	-0.0222	0.0053
rs4061660	Yes	SMG6	A	G	0.1667	0.0137	0.0046
rs4411908	Yes	GBE1	C	A	0.3750	0.0150	0.0038
rs4946932	Yes	FOXO3	C	A	0.7000	0.0163	0.0041
rs571312	Yes	MC4R	A	C	0.2833	0.0553	0.0036
rs6096969	Yes	ZFP64	G	A	0.7250	0.0202	0.0042
rs6604872	Yes	FPGT-TNNI3K	T	C	0.4250	0.0237	0.0031
rs6785875	Yes	FHIT	T	C	0.6667	0.0186	0.0038
rs943005	Yes	TFAP2B	T	C	0.1000	0.0443	0.0040
rs9957264	Yes	GRP	A	C	0.1333	-0.0229	0.0049

[a]As classified by the GIANT consortium.

[b]Reported by the GIANT consortium, which extracted from the HapMap Phase II CEU reference panel.

Supplementary Table S4. SNP-bipolar disorder regression coefficients corresponding to per-allele log odds ratio (logOR) and associated standard errors (SE) obtained from the PGC consortium for 97 BMI-associated SNPs reported by the GIANT consortium.

SNP	Effect allele	Other allele	logOR	SE	Studentized residuals		Cook's distance	
					IVW	MR-Egger	IVW	MR-Egger
rs1000940	G	A	-0.03082	0.0274	-0.83552	-0.74641	0.00374	0.00354
rs10132280	A	C	0.04488	0.0256	-1.32825	-1.26864	0.01540	0.00965
rs1016287	T	C	0.00864	0.0259	0.34012	0.40277	0.00099	0.00097
rs10182181	A	G	0.01242	0.0244	-0.29893	-0.31353	0.00157	0.00087
rs10733682	A	G	0.01074	0.0256	0.39059	0.50981	0.00077	0.00219
rs10938397	A	G	-0.00904	0.0245	0.43739	0.32741	0.00578	0.00223
rs10968576	G	A	0.03366	0.0251	1.15861	1.20542	0.01438	0.00898
rs11030104	A	G	0.00975	0.0288	0.39407	0.29032	0.00358	0.00139
rs11057405	A	G	-0.13216	0.0469	2.35493	2.34980	0.02463	0.01238
rs11126666	G	A	0.02163	0.0265	-0.58245	-0.50323	0.00227	0.00157
rs11165643	C	T	-0.02706	0.0240	0.97775	1.06061	0.00860	0.00802
rs11191560	T	C	0.14618	0.0422	-2.79349	-2.80733	0.04219	0.02152
rs11583200	C	T	0.01242	0.0241	0.47165	0.59602	0.00131	0.00329
rs1167827	A	G	-0.00451	0.0289	0.18303	0.26224	0.00018	0.00037
rs11688816	A	G	0.04593	0.0240	-1.47335	-1.35481	0.01197	0.01762
rs11727676	C	T	-0.07279	0.0701	0.86893	0.84664	0.00214	0.00118
rs11847697	T	C	0.06283	0.0648	0.83666	0.76222	0.00440	0.00331
rs12016871	C	T	0.02946	0.0302	-0.69343	-0.69816	0.00514	0.00261
rs12286929	G	A	-0.00070	0.0231	0.05574	0.13820	0.00003	0.00015
rs12401738	A	G	-0.08078	0.0245	-2.63607	-2.55524	0.05287	0.04363
rs12429545	G	A	-0.03276	0.0387	0.74754	0.72186	0.00456	0.00228
rs12446632	A	G	0.00886	0.0341	-0.10637	-0.18754	0.00018	0.00037
rs12566985	G	A	0.01147	0.0234	0.47826	0.53398	0.00270	0.00205
rs12885454	C	A	0.00924	0.0246	0.36995	0.45905	0.00106	0.00152
rs12940622	A	G	0.04162	0.0234	-1.35823	-1.24595	0.01203	0.01450
rs13021737	A	G	-0.01532	0.0306	0.57499	0.32916	0.01448	0.00576
rs13078960	T	G	-0.03701	0.0290	1.10910	1.10668	0.01407	0.00701
rs13107325	C	T	-0.06539	0.0456	1.23968	1.14305	0.01829	0.01358
rs13191362	A	G	-0.00572	0.0351	-0.06227	-0.05095	0.00003	0.00001
rs13201877	A	G	-0.00290	0.0342	0.12508	0.16898	0.00008	0.00010
rs1441264	A	G	-0.03005	0.0253	-0.88661	-0.77470	0.00409	0.00511
rs1460676	T	C	0.02235	0.0318	-0.50508	-0.43128	0.00107	0.00085
rs1516725	T	C	-0.05704	0.0353	1.41289	1.30625	0.03548	0.02459
rs1528435	T	C	0.02573	0.0242	0.90951	1.03663	0.00487	0.00973
rs1558902	A	T	-0.01056	0.0237	-0.06467	-0.78165	0.00062	0.19454
rs16851483	G	T	0.08883	0.0503	-1.33437	-1.43824	0.01778	0.01822
rs16907751	C	T	-0.14920	0.0641	-1.82871	-1.85434	0.01055	0.00612
rs16951275	C	T	0.03034	0.0282	-0.76479	-0.78159	0.00782	0.00415
rs17001654	C	G	0.00459	0.0332	-0.03153	-0.04127	0.00001	0.00001
rs17024393	C	T	-0.03532	0.0769	-0.29211	-0.41320	0.00069	0.00174
rs17094222	C	T	-0.03232	0.0289	-0.81757	-0.78047	0.00542	0.00286
rs17203016	G	A	0.04353	0.0308	1.18707	1.25928	0.00708	0.00705
rs17405819	C	T	-0.05077	0.0252	1.70076	1.77774	0.02443	0.01957
rs17724992	A	G	-0.04041	0.0266	-1.15078	-1.06145	0.00763	0.00746
rs1808579	T	C	0.05003	0.0244	-1.58826	-1.46739	0.01263	0.02079

rs1928295	C	T	0.00965	0.0234	-0.25929	-0.14857	0.00048	0.00020
rs2033529	G	A	-0.03508	0.0270	-0.97532	-0.88350	0.00512	0.00517
rs2033732	C	T	-0.00702	0.0274	-0.14389	-0.05252	0.00011	0.00002
rs205262	A	G	-0.01949	0.0266	0.65353	0.72344	0.00323	0.00302
rs2075650	A	G	0.04793	0.0404	0.99965	1.02268	0.00442	0.00252
rs2080454	A	C	-0.05003	0.0252	1.65360	1.79667	0.01296	0.02861
rs2112347	G	T	-0.00280	0.0242	0.18401	0.21733	0.00044	0.00033
rs2121279	T	C	-0.02567	0.0355	-0.51546	-0.48238	0.00139	0.00072
rs2176040	G	A	0.00521	0.0243	-0.12083	0.03483	0.00005	0.00002
rs2176598	T	C	0.03170	0.0273	0.98731	1.07978	0.00557	0.00712
rs2207139	G	A	0.01076	0.0308	0.40388	0.28057	0.00383	0.00148
rs2245368	T	C	-0.02274	0.0377	0.55107	0.53572	0.00236	0.00114
rs2287019	C	T	-0.03999	0.0399	-0.72141	-0.76477	0.00464	0.00305
rs2365389	C	T	-0.00300	0.0239	-0.02876	0.06837	0.00001	0.00004
rs2650492	A	G	0.02068	0.0302	0.60226	0.67553	0.00186	0.00217
rs2820292	A	C	0.04669	0.0234	-1.53177	-1.43224	0.01750	0.01732
rs2836754	C	T	0.04229	0.0242	1.45899	1.61048	0.01049	0.02602
rs29941	A	G	-0.03604	0.0248	1.22440	1.34717	0.00873	0.01502
rs3101336	T	C	-0.00429	0.0242	0.25969	0.21766	0.00143	0.00054
rs3736485	A	G	-0.02133	0.0233	-0.66350	-0.54211	0.00275	0.00295
rs3810291	A	G	-0.06422	0.0275	-1.79974	-1.79214	0.03664	0.01828
rs3817334	C	T	0.01607	0.0237	-0.44616	-0.41395	0.00269	0.00124
rs3849570	A	C	0.03864	0.0243	1.33978	1.45939	0.01159	0.01749
rs3888190	A	C	0.04507	0.0238	1.64506	1.62947	0.04933	0.02453
rs4256980	G	C	0.06732	0.0245	2.31657	2.42055	0.04069	0.03982
rs4740619	T	C	-0.02323	0.0237	-0.71531	-0.59918	0.00319	0.00339
rs4787491	A	G	0.03227	0.0247	-0.98605	-0.85708	0.00437	0.00754
rs492400	T	C	0.00481	0.0235	-0.10533	0.03772	0.00006	0.00002
rs543874	G	A	-0.00110	0.0294	0.11068	-0.05149	0.00037	0.00007
rs6091540	C	T	-0.01694	0.0267	-0.44392	-0.34770	0.00107	0.00084
rs6465468	G	T	-0.01126	0.0275	0.37595	0.49418	0.00056	0.00190
rs6477694	C	T	-0.00090	0.0245	0.03107	0.15286	0.00001	0.00022
rs6567160	C	T	-0.00783	0.0275	-0.05546	-0.30593	0.00014	0.00482
rs657452	A	G	-0.00280	0.0245	-0.01222	0.05502	0.00000	0.00002
rs6804842	A	G	0.02010	0.0237	-0.60774	-0.49710	0.00246	0.00223
rs7138803	G	A	0.01796	0.0249	-0.46803	-0.49066	0.00389	0.00219
rs7141420	T	C	-0.01961	0.0231	-0.58956	-0.52801	0.00396	0.00207
rs7164727	T	C	-0.01400	0.0252	-0.38032	-0.27040	0.00081	0.00061
rs7239883	G	A	-0.02010	0.0239	-0.60967	-0.47912	0.00191	0.00242
rs7243357	G	T	-0.01990	0.0320	0.55128	0.61206	0.00153	0.00151
rs758747	C	T	0.00270	0.0300	-0.00768	0.04878	0.00000	0.00001
rs7599312	G	A	-0.01430	0.0264	-0.35940	-0.29134	0.00099	0.00050
rs7715256	G	T	-0.01136	0.0236	-0.32341	-0.18812	0.00055	0.00039
rs7899106	A	G	0.05629	0.0559	-0.74063	-0.78774	0.00299	0.00224
rs7903146	T	C	-0.03768	0.0259	1.24231	1.30358	0.01366	0.00987
rs9374842	T	C	0.03314	0.0278	1.00746	1.10923	0.00499	0.00782
rs9400239	C	T	-0.02861	0.0256	-0.82690	-0.72793	0.00402	0.00398
rs9540493	G	A	-0.01045	0.0259	0.37625	0.49595	0.00068	0.00206
rs9641123	G	C	-0.01430	0.0250	0.51922	0.62450	0.00172	0.00301
rs977747	T	G	-0.05269	0.0239	-1.71461	-1.59172	0.01528	0.02542
rs9914578	G	C	-0.01696	0.0291	-0.40375	-0.32619	0.00085	0.00057
rs9925964	G	A	0.06989	0.0241	-2.29455	-2.19535	0.03481	0.03803

Supplementary Table S5. SNP-schizophrenia regression coefficients corresponding to per-allele log odds ratio (logOR) and associated standard errors (SE) obtained from the PGC consortium for 96 BMI-associated SNPs reported by the GIANT consortium.

SNP	Effect allele	Other allele	logOR	SE	Studentized residuals		Cook's distance	
					IVW	MR-Egger	IVW	MR-Egger
rs1000940	G	A	-0.01780	0.0114	-0.71030	-0.56778	0.00313	0.00237
rs10132280	A	C	0.01890	0.0117	-0.73323	-0.64928	0.00455	0.00244
rs1016287	T	C	0.00510	0.0116	0.22765	0.32358	0.00044	0.00062
rs10182181	A	G	0.00260	0.0106	-0.08155	-0.10673	0.00012	0.00011
rs10733682	A	G	-0.00570	0.0106	-0.23169	-0.04317	0.00032	0.00002
rs10938397	A	G	0.01600	0.0107	-0.66366	-0.85497	0.01393	0.01601
rs10968576	G	A	0.02900	0.0114	1.22243	1.30134	0.01548	0.01007
rs11030104	A	G	0.04920	0.0129	1.86233	1.71692	0.07690	0.04754
rs11057405	A	G	-0.06350	0.0196	1.54645	1.54749	0.01256	0.00636
rs11126666	G	A	0.01160	0.0119	-0.43479	-0.31684	0.00125	0.00062
rs11165643	C	T	0.02360	0.0108	-1.00173	-0.89392	0.00891	0.00562
rs11191560	T	C	0.14750	0.0182	-4.07595	-4.14326	0.08880	0.04620
rs11583200	C	T	0.03850	0.0109	1.68935	1.90783	0.01603	0.03185
rs1167827	A	G	-0.01020	0.0110	0.45395	0.60129	0.00152	0.00268
rs11688816	A	G	0.02600	0.0107	-1.12106	-0.94238	0.00703	0.00866
rs11727676	C	T	-0.00500	0.0195	0.14051	0.08375	0.00015	0.00003
rs11847697	T	C	0.02760	0.0281	0.47836	0.36128	0.00154	0.00080
rs12286929	G	A	0.00910	0.0106	0.42455	0.55189	0.00166	0.00225
rs12401738	A	G	-0.03300	0.0115	-1.32929	-1.22102	0.01284	0.00947
rs12429545	G	A	-0.01010	0.0155	0.32909	0.28556	0.00111	0.00045
rs12446632	A	G	0.02120	0.0157	-0.60365	-0.73381	0.00530	0.00541
rs12566985	G	A	0.02270	0.0107	1.02246	1.11507	0.01167	0.00842
rs12885454	C	A	0.03510	0.0111	1.51403	1.67064	0.01711	0.01919
rs12940622	A	G	0.00500	0.0106	-0.20015	-0.02411	0.00026	0.00001
rs13021737	A	G	-0.01370	0.0141	0.51090	0.13635	0.01074	0.00094
rs13078960	T	G	-0.00630	0.0136	0.24141	0.23727	0.00061	0.00030
rs13107325	C	T	-0.15190	0.0215	3.53995	3.41671	0.12007	0.09934
rs13191362	A	G	-0.00060	0.0168	0.00220	0.01752	0.00000	0.00000
rs13201877	A	G	-0.00180	0.0155	0.07131	0.13730	0.00003	0.00006
rs1441264	A	G	0.01250	0.0109	0.55358	0.74861	0.00173	0.00515
rs1460676	T	C	0.01290	0.0139	-0.41617	-0.30340	0.00076	0.00044
rs1516725	T	C	-0.01530	0.0162	0.47495	0.31128	0.00387	0.00136
rs1528435	T	C	0.00820	0.0109	0.36928	0.55588	0.00080	0.00278
rs1558902	A	T	-0.00630	0.0109	-0.19529	-1.28554	0.00534	0.48663
rs16851483	G	T	0.00070	0.0207	0.01094	-0.14835	0.00000	0.00024
rs16907751	C	T	-0.03420	0.0170	-0.91943	-0.98800	0.00782	0.00513
rs16951275	C	T	-0.01950	0.0126	0.75431	0.73512	0.00762	0.00369
rs17001654	C	G	0.03640	0.0155	-1.07966	-1.10552	0.00988	0.00523
rs17024393	C	T	0.08220	0.0304	1.29885	1.10814	0.01712	0.01617
rs17094222	C	T	-0.00340	0.0129	-0.10102	-0.04475	0.00008	0.00001
rs17203016	G	A	0.02550	0.0134	0.90839	1.02534	0.00441	0.00495
rs17405819	C	T	-0.02190	0.0114	0.92280	1.03732	0.00717	0.00662
rs17724992	A	G	0.01050	0.0121	0.42317	0.56844	0.00101	0.00208
rs1808579	T	C	-0.01020	0.0106	0.46687	0.67949	0.00118	0.00482
rs1928295	C	T	0.01920	0.0106	-0.82723	-0.66852	0.00470	0.00392

rs2033529	G	A	0.01260	0.0117	0.52102	0.67891	0.00157	0.00326
rs2033732	C	T	-0.01470	0.0121	-0.54875	-0.41310	0.00166	0.00112
rs205262	A	G	-0.00500	0.0119	0.21733	0.32242	0.00036	0.00060
rs2075650	A	G	-0.00360	0.0156	-0.08852	-0.05198	0.00005	0.00001
rs2080454	A	C	0.03040	0.0109	-1.29291	-1.11336	0.00856	0.01201
rs2112347	G	T	0.01980	0.0109	-0.82491	-0.78341	0.00856	0.00414
rs2121279	T	C	-0.02630	0.0160	-0.75042	-0.70686	0.00288	0.00151
rs2176040	G	A	0.00060	0.0111	-0.01066	0.22450	0.00000	0.00061
rs2176598	T	C	0.02320	0.0123	0.90096	1.04563	0.00458	0.00657
rs2207139	G	A	-0.00800	0.0138	-0.23509	-0.43485	0.00129	0.00359
rs2245368	T	C	0.00580	0.0155	-0.15133	-0.18045	0.00021	0.00015
rs2287019	C	T	-0.01830	0.0138	-0.59185	-0.68360	0.00526	0.00414
rs2365389	C	T	-0.03500	0.0108	-1.50793	-1.37658	0.01674	0.01450
rs2650492	A	G	0.00360	0.0127	0.15084	0.26768	0.00013	0.00039
rs2820292	A	C	0.00450	0.0107	-0.17527	-0.02141	0.00022	0.00000
rs2836754	C	T	0.01630	0.0109	0.71566	0.93272	0.00253	0.00877
rs29941	A	G	-0.00620	0.0113	0.27410	0.44612	0.00043	0.00161
rs3101336	T	C	-0.01830	0.0111	0.81082	0.75288	0.01316	0.00614
rs3736485	A	G	-0.02570	0.0108	-1.09693	-0.92575	0.00693	0.00793
rs3810291	A	G	-0.02690	0.0115	-1.07210	-1.06835	0.01521	0.00759
rs3817334	C	T	0.00390	0.0107	-0.14226	-0.09535	0.00027	0.00006
rs3849570	A	C	0.00850	0.0111	0.37667	0.54391	0.00089	0.00237
rs3888190	A	C	0.01960	0.0111	0.86254	0.84385	0.01270	0.00617
rs4256980	G	C	-0.00150	0.0110	-0.04182	0.08756	0.00001	0.00005
rs4740619	T	C	-0.01510	0.0107	-0.64003	-0.46674	0.00251	0.00202
rs4787491	A	G	0.05110	0.0106	-2.28930	-2.09880	0.02450	0.04737
rs492400	T	C	-0.00430	0.0107	0.20402	0.42443	0.00020	0.00200
rs543874	G	A	-0.00900	0.0134	-0.27500	-0.52996	0.00220	0.00738
rs6091540	C	T	-0.02400	0.0117	-0.94169	-0.79957	0.00498	0.00458
rs6465468	G	T	0.02020	0.0122	-0.75763	-0.58979	0.00231	0.00275
rs6477694	C	T	0.02200	0.0110	0.95435	1.15769	0.00495	0.01209
rs6567160	C	T	0.01770	0.0125	0.72603	0.37126	0.02365	0.00694
rs657452	A	G	0.03530	0.0109	1.55510	1.67996	0.02253	0.01851
rs6804842	A	G	0.01750	0.0107	-0.74454	-0.58181	0.00362	0.00299
rs7138803	G	A	-0.01010	0.0109	0.46814	0.43599	0.00406	0.00181
rs7141420	T	C	0.02230	0.0105	1.02333	1.12969	0.01145	0.00903
rs7164727	T	C	-0.00920	0.0114	-0.35823	-0.19390	0.00070	0.00030
rs7239883	G	A	0.02950	0.0108	1.30111	1.53500	0.00841	0.02382
rs7243357	G	T	0.01960	0.0137	-0.64985	-0.56231	0.00231	0.00139
rs758747	C	T	-0.00970	0.0124	0.38588	0.48314	0.00108	0.00122
rs7599312	G	A	-0.03520	0.0121	-1.34854	-1.25872	0.01297	0.00872
rs7715256	G	T	-0.03920	0.0109	-1.68259	-1.49913	0.01348	0.02252
rs7899106	A	G	0.05200	0.0260	-0.91829	-0.99652	0.00423	0.00334
rs7903146	T	C	-0.01090	0.0121	0.44276	0.53004	0.00161	0.00151
rs9374842	T	C	0.00420	0.0124	0.17504	0.32314	0.00015	0.00067
rs9400239	C	T	-0.06090	0.0115	-2.53063	-2.39689	0.03518	0.04044
rs9540493	G	A	0.00230	0.0110	-0.07965	0.10731	0.00003	0.00011
rs9641123	G	C	-0.00870	0.0109	0.39249	0.55818	0.00104	0.00253
rs977747	T	G	-0.02200	0.0110	-0.91824	-0.73497	0.00423	0.00522
rs9914578	G	C	-0.04190	0.0132	-1.47787	-1.37334	0.01086	0.00955
rs9925964	G	A	0.01350	0.0110	-0.55302	-0.40339	0.00204	0.00129

Supplementary Table S6. SNP-major depressive disorder regression coefficients corresponding to per-allele log odds ratio (logOR) and associated standard errors (SE) obtained from the PGC consortium for 90[a] BMI-associated SNPs reported by the GIANT consortium.

SNP	Effect allele	Other allele	logOR	SE	Studentized residuals		Cook's distance	
					IVW	MR-Egger	IVW	MR-Egger
rs1000940	G	A	-0.03150	0.0233	-1.25354	-1.21408	0.01000	0.01041
rs10132280	A	C	0.00200	0.0241	-0.18398	-0.16164	0.00029	0.00015
rs1016287	T	C	0.00100	0.0234	-0.08039	-0.05738	0.00006	0.00002
rs10182181	A	G	0.00200	0.0214	-0.25147	-0.25920	0.00126	0.00068
rs10464483	C	T	-0.00300	0.0219	0.01637	0.06022	0.00000	0.00003
rs10513801	G	T	-0.02567	0.0318	0.51968	0.47289	0.00516	0.00355
rs10733682	A	G	-0.00250	0.0213	-0.19672	-0.14802	0.00025	0.00022
rs10808859	A	T	0.00399	0.0260	0.03387	0.06099	0.00001	0.00002
rs10838184	C	G	0.02664	0.0249	0.81083	0.83975	0.00422	0.00405
rs10840100	G	A	0.04479	0.0220	1.63647	1.66799	0.02241	0.01968
rs10968576	G	A	0.04688	0.0230	1.62431	1.63366	0.02876	0.01620
rs11030104	A	G	-0.03532	0.0257	-1.38218	-1.43202	0.04712	0.03751
rs11126666	G	A	-0.03633	0.0237	1.20412	1.23361	0.01038	0.00948
rs11165643	C	T	0.02819	0.0216	-1.23768	-1.20476	0.01467	0.01042
rs11191560	T	C	0.11154	0.0376	-2.71585	-2.70958	0.04354	0.02218
rs11583200	C	T	0.02378	0.0218	0.83002	0.87832	0.00430	0.00705
rs11625899	T	C	-0.01349	0.0226	0.40111	0.43664	0.00119	0.00139
rs11672660	C	T	-0.02078	0.0298	-0.73265	-0.74821	0.00686	0.00409
rs1167827	A	G	0.00200	0.0241	-0.16996	-0.13719	0.00019	0.00012
rs11688816	A	G	-0.01511	0.0215	0.50053	0.55124	0.00152	0.00299
rs12286929	G	A	-0.02859	0.0212	-1.27723	-1.24336	0.01606	0.01155
rs12369179	T	C	-0.01025	0.0422	0.11931	0.11453	0.00007	0.00003
rs12401738	A	G	-0.01349	0.0222	-0.62961	-0.59668	0.00341	0.00252
rs12429545	G	A	0.01572	0.0321	-0.54039	-0.55186	0.00302	0.00173
rs12446632	A	G	0.01784	0.0305	-0.65730	-0.69226	0.00723	0.00570
rs12641981	C	T	-0.01686	0.0216	0.44718	0.39896	0.00666	0.00374
rs12940622	A	G	0.01094	0.0212	-0.54005	-0.49466	0.00204	0.00229
rs13012571	C	T	-0.01784	0.0282	0.28902	0.18991	0.00370	0.00198
rs13098327	G	A	-0.01094	0.0267	0.21601	0.21211	0.00055	0.00026
rs13191362	A	G	0.00995	0.0323	0.15882	0.16082	0.00018	0.00009
rs1421085	C	T	0.04103	0.0218	1.23945	1.19641	0.22878	0.47600
rs1441264	A	G	0.02762	0.0220	0.97297	1.02222	0.00565	0.00950
rs1460676	T	C	0.02176	0.0288	-0.72248	-0.69179	0.00231	0.00215
rs1462433	A	G	-0.07505	0.0232	2.72113	2.72315	0.08033	0.04286
rs1515104	A	T	0.00598	0.0222	-0.31873	-0.27014	0.00056	0.00069
rs1528435	T	C	0.00499	0.0218	0.09653	0.14284	0.00006	0.00019
rs16851483	G	T	0.00884	0.0444	-0.29883	-0.33994	0.00101	0.00120
rs17001654	C	G	-0.03201	0.0301	0.78325	0.77367	0.00602	0.00300
rs17094222	C	T	0.03521	0.0283	0.95398	0.96240	0.00666	0.00377
rs17203016	G	A	0.00965	0.0273	0.20757	0.23291	0.00024	0.00025
rs17724992	A	G	-0.01908	0.0252	-0.73455	-0.69919	0.00303	0.00294
rs1808579	T	C	0.02567	0.0212	-1.12643	-1.07625	0.00740	0.01214
rs1885988	T	C	-0.04458	0.0273	1.29817	1.31676	0.01033	0.00765
rs1928295	C	T	0.03916	0.0215	-1.67247	-1.62872	0.01978	0.02240
rs1978487	C	T	0.01816	0.0225	-0.78341	-0.74094	0.00389	0.00446

rs2033529	G	A	0.01248	0.0237	0.35049	0.38802	0.00075	0.00106
rs205262	A	G	0.02274	0.0238	-0.92458	-0.89610	0.00697	0.00472
rs2075650	A	G	0.00995	0.0346	0.15466	0.16159	0.00013	0.00007
rs2080454	A	C	-0.00220	0.0225	-0.00597	0.04290	0.00000	0.00002
rs2121279	T	C	0.01784	0.0322	0.37862	0.38895	0.00079	0.00048
rs2241420	A	G	0.04497	0.0256	-1.65335	-1.64814	0.03331	0.01659
rs2650492	A	G	-0.02573	0.0254	-0.95938	-0.92774	0.00577	0.00470
rs2820292	A	C	0.00797	0.0216	-0.42041	-0.38083	0.00137	0.00119
rs2836754	C	T	0.01582	0.0221	0.51835	0.57144	0.00140	0.00326
rs29941	A	G	-0.03604	0.0233	1.22653	1.26940	0.00860	0.01224
rs3101336	T	C	-0.03936	0.0220	1.35849	1.33305	0.04035	0.02142
rs3736485	A	G	0.02078	0.0214	0.72644	0.77605	0.00338	0.00577
rs3797580	G	A	-0.02956	0.0222	1.00311	1.01600	0.01181	0.00683
rs3810291	A	G	-0.00431	0.0243	-0.28977	-0.28704	0.00109	0.00054
rs3817334	C	T	0.02041	0.0217	-0.94805	-0.93328	0.01246	0.00631
rs3888190	A	C	0.01489	0.0218	0.41251	0.40113	0.00329	0.00160
rs3904532	A	G	0.03343	0.0305	-1.01989	-0.99635	0.00519	0.00352
rs4061660	A	G	0.01784	0.0263	0.51204	0.56654	0.00067	0.00289
rs4411908	C	A	-0.01686	0.0221	-0.72793	-0.67319	0.00231	0.00514
rs4740619	T	C	-0.01106	0.0214	-0.53802	-0.49187	0.00193	0.00227
rs4787491	A	G	0.02469	0.0221	-1.03698	-0.98582	0.00524	0.01008
rs492400	T	C	-0.00844	0.0217	0.24236	0.29835	0.00030	0.00098
rs4946932	C	A	-0.02274	0.0232	-0.91691	-0.86873	0.00391	0.00685
rs543874	G	A	0.02727	0.0268	0.65691	0.59299	0.01357	0.01030
rs571312	A	C	-0.05351	0.0247	-2.20287	-2.37561	0.22918	0.30074
rs6096969	G	A	0.02522	0.0235	0.80930	0.84169	0.00458	0.00466
rs6465468	G	T	-0.01882	0.0242	0.57733	0.62446	0.00149	0.00318
rs6477694	C	T	0.00250	0.0220	0.00221	0.04968	0.00000	0.00002
rs657452	A	G	0.04497	0.0220	1.63502	1.65659	0.02644	0.01824
rs6604872	T	C	0.03633	0.0215	1.31545	1.33302	0.01976	0.01237
rs6785875	T	C	-0.08687	0.0221	-3.68283	-3.63463	0.07940	0.09579
rs6804842	A	G	-0.00602	0.0214	0.13526	0.17938	0.00013	0.00029
rs7138803	G	A	-0.00300	0.0219	-0.05572	-0.06751	0.00006	0.00005
rs7141420	T	C	0.02274	0.0211	0.78462	0.80496	0.00727	0.00475
rs7164727	T	C	-0.03874	0.0229	-1.54582	-1.50186	0.01370	0.01789
rs7239883	G	A	0.02327	0.0215	0.82827	0.88441	0.00377	0.00822
rs7599312	G	A	-0.02469	0.0238	-0.99467	-0.96559	0.00798	0.00549
rs7715256	G	T	-0.00499	0.0214	-0.28794	-0.23515	0.00046	0.00060
rs7899106	A	G	0.02469	0.0493	-0.52006	-0.53860	0.00165	0.00122
rs7903146	T	C	0.00399	0.0233	-0.26506	-0.24325	0.00068	0.00036
rs9374842	T	C	0.02664	0.0249	0.81909	0.85580	0.00358	0.00473
rs943005	T	C	0.01489	0.0283	0.26141	0.21031	0.00162	0.00086
rs9540493	G	A	0.01542	0.0219	-0.69112	-0.64346	0.00279	0.00392
rs977747	T	G	0.04402	0.0215	1.66582	1.72482	0.01547	0.02969
rs9957264	A	C	-0.02143	0.0291	0.53043	0.54680	0.00166	0.00116

[a]62 were the same variants and 28 were proxies for missing variants in the major depressive disorder data set.

Supplementary Table S7. Classification of SNPs into 16 biological categories as defined by the GIANT consortium.

Biological category	SNPs
Neuronal developmental processes	rs3101336, rs11165643, rs2820292, rs492400, rs6804842, rs13078960, rs2033529, rs9400239, rs13201877, rs13191362, rs4740619, rs10733682, rs17094222, rs4256980, rs12286929, rs7138803, rs11057405, rs12885454, rs7141420, rs3736485, rs16951275, rs3888190, rs4787491, rs2080454, rs7239883, rs29941, rs2287019, rs3810291, rs2836754, rs13098327[a], rs4946932[a], rs3904532[a], rs10840100[a], rs12369179[a], rs11625899[a], rs2241420[a], rs11672660[a]
Neurotransmission	rs13191362, rs3736485, rs4787491, rs10938397, rs1167827, rs7899106, rs9540493, rs10132280, rs9925964, rs9914578, rs12641981[a], rs1978487[a], rs4061660[a]
Hypothalamic expression and regulatory function	rs4256980, rs3736485, rs3888190, rs10938397, rs13021737, rs10182181, rs1516725, rs17405819, rs11030104, rs7164727, rs1558902, rs7243357, rs6567160, rs10840100[a], rs12641981[a], rs13012571[a], rs10513801[a], rs1462433[a], rs1421085[a], rs9957264[a], rs571312[a]
Neuronal expression.	rs13191362, rs9914578, rs10182181, rs11583200, rs12401738, rs11126666, rs7599312, rs3849570, rs10968576, rs12446632, rs1808579, rs2075650, rs4061660[a], rs4411908[a]
Lipid biosynthesis and metabolismo	rs492400, rs4787491, rs7164727, rs1808579, rs2075650, rs2112347, rs1928295, rs11191560, rs3817334, rs2650492, rs3797580[a]
Bone development	rs6804842, rs16951275, rs1167827, rs16851483, rs11727676, rs205262, rs9641123, rs17724992, rs6091540, rs2241420[a], rs10464483[a], rs6096969[a]
Mitogen activated protein kinase1/Extracellular signal-regulated kinases	rs4256980, rs16951275, rs4787491, rs7239883, rs7243357, rs3817334, rs16851483, rs12566985, rs12940622, rs10840100[a], rs2241420[a], rs9957264[a], rs6604872[a]
Endocytosis/Exocytosis	rs13191362, rs17094222, rs11057405, rs3736485, rs7239883, rs1167827, rs10132280, rs9925964, rs1808579, rs543874, rs11688816, rs2121279, rs17001654, rs1000940, rs12369179[a], rs1978487[a]
Tumorigenesis	rs4256980, rs7138803, rs3736485, rs4787491, rs2836754, rs7599312, rs3817334, rs16851483, rs12429545, rs11688816, rs2121279, rs10840100[a]
Apoptosis	rs2033529, rs13191362, rs4740619, rs17094222, rs11057405, rs2836754, rs1167827, rs9925964, rs3817334, rs2650492, rs1000940, rs2365389, rs758747, rs12369179[a], rs1978487[a], rs6785875[a]
Membrane Proteins	rs2033529, rs29941, rs3810291, rs1167827, rs13021737, rs6567160, rs7599312, rs2075650, rs2112347, rs3817334, rs1000940, rs13012571[a], rs571312[a], rs3797580[a]
Monogenic obesity and/or Energy homeostasis	rs4256980, rs3736485, rs3888190, rs10182181, rs11030104, rs7164727, rs1558902, rs6567160, rs6465468, rs10840100 [a], rs1421085 [a], rs571312[a]
Immune System	rs13078960, rs13201877, rs17094222, rs12286929, rs12885454, rs9925964, rs12401738, rs2075650, rs2112347, rs1928295, rs3817334, rs9641123, rs758747, rs9374842, rs11847697, rs13098327 [a], rs3904532 [a], rs11625899 [a], rs1978487 [a], rs3797580[a], rs10464483[a]
Glucose homeostasis and/or diabetes	rs9400239, rs2287019, rs7164727, rs6567160, rs11583200, rs3849570, rs3817334, rs12940622, rs17203016, rs7903146, rs2176040, rs4946932[a], rs11672660[a], rs571312[a], rs4411908[a], rs1515104[a]
Cell cycle	rs492400, rs4256980, rs11057405, rs12885454, rs4787491, rs1167827, rs9925964, rs9914578, rs10182181, rs7164727, rs1558902, rs12401738, rs1808579, rs2112347, rs1000940, rs758747, rs977747, rs9374842, rs11847697, rs1016287, rs657452, rs1460676, rs2245368, rs10840100[a], rs12369179[a], rs11625899[a], rs1978487[a], rs4061660[a], rs1421085[a], rs3797580[a]
Prioritized by GRAIL-Putative coding variant annotation-CNV-eQTL-DEPICT but not in above categories	rs492400, rs4740619, rs7239883, rs2287019, rs3810291, rs1167827, rs9540493, rs9925964, rs7164727, rs12446632, rs1808579, rs11191560, rs3817334, rs205262, rs17724992, rs12566985, rs12016871, rs9374842, rs1016287, rs2176040, rs7715256, rs16907751, rs2033732, rs6477694, rs1441264, rs11672660[a], rs1978487[a], rs6604872[a], rs1885988[a], rs1515104[a], rs10808859[a]

[a]Proxy SNPs used in major depressive disorder analyses.
